# A stimulus-dependent spike threshold is an optimal neural coder

**DOI:** 10.3389/fncom.2015.00061

**Published:** 2015-06-02

**Authors:** Douglas L. Jones, Erik C. Johnson, Rama Ratnam

**Affiliations:** ^1^Department of Electrical and Computer Engineering, University of Illinois at Urbana-ChampaignUrbana, IL, USA; ^2^Beckman Institute for Advanced Science and Technology, University of Illinois at Urbana-ChampaignUrbana, IL, USA; ^3^Coordinated Science Laboratory, University of Illinois at Urbana-ChampaignUrbana, IL, USA; ^4^Advanced Digital Sciences Center, Illinois at Singapore Pte. Ltd., SingaporeSingapore

**Keywords:** neural coding, coding fidelity, energy-efficient coding, dynamic threshold, spike-timing, source coding, spike-threshold

## Abstract

A neural code based on sequences of spikes can consume a significant portion of the brain's energy budget. Thus, energy considerations would dictate that spiking activity be kept as low as possible. However, a high spike-rate improves the coding and representation of signals in spike trains, particularly in sensory systems. These are competing demands, and selective pressure has presumably worked to optimize coding by apportioning a minimum number of spikes so as to maximize coding fidelity. The mechanisms by which a neuron generates spikes while maintaining a fidelity criterion are not known. Here, we show that a signal-dependent neural threshold, similar to a dynamic or adapting threshold, optimizes the trade-off between spike generation (encoding) and fidelity (decoding). The threshold mimics a post-synaptic membrane (a low-pass filter) and serves as an internal decoder. Further, it sets the average firing rate (the energy constraint). The decoding process provides an internal copy of the coding error to the spike-generator which emits a spike when the error equals or exceeds a spike threshold. When optimized, the trade-off leads to a deterministic spike firing-rule that generates optimally timed spikes so as to maximize fidelity. The optimal coder is derived in closed-form in the limit of high spike-rates, when the signal can be approximated as a piece-wise constant signal. The predicted spike-times are close to those obtained experimentally in the primary electrosensory afferent neurons of weakly electric fish (*Apteronotus leptorhynchus*) and pyramidal neurons from the somatosensory cortex of the rat. We suggest that KCNQ/Kv7 channels (underlying the M-current) are good candidates for the decoder. They are widely coupled to metabolic processes and do not inactivate. We conclude that the neural threshold is optimized to generate an energy-efficient and high-fidelity neural code.

## Introduction

The coding and representation of signals using sequences of action potentials or spikes is an energy intensive process (Attwell and Laughlin, 2001). It imposes a significant cost on information transmission (Laughlin et al., 1998) and exerts selective pressure on the nervous system to generate energy-efficient codes (Niven and Laughlin, 2008). However, lowering energy consumption (spike-rate) can adversely affect sensory information processing, particularly for maintaining coding fidelity. How then does a neuron balance energy-consumption and fidelity? One possibility is that for a given mean spike-rate the optimum trade-off is achieved by timing the spikes to produce the best possible reconstruction of the input signal. This would require a reconstruction mechanism (a decoder) to reside within the neuron so that an internal error signal can be generated by comparing the reconstruction with the input signal. The error feedback to the spike generator (the encoder) will allow the neuron to optimally time its spikes so as to minimize the error. This is similar to certain forms of digital coding where the decoder is built into the coder so that an internal copy of the error is available (Jayant and Noll, 1984).

Traditionally, the neural coding literature has treated the encoding (spike-generation) problem and the decoding (reconstruction) problem as separate and distinct processes. More importantly, from the perspective of this work, the encoder and decoder mechanisms have never been considered as embodied within the same neuron or as sharing coupled biophysical mechanisms. Consequently reconstruction has proceeded on the assumption that the encoding process is separate and unknown (for e.g., see Eggermont et al., 1983; de Ruyter van Steveninck and Bialek, 1988; Gabbiani, 1996). This work reconciles encoding and decoding and situates both processes squarely within the biophysical capabilities of the same neuron. In essence, we claim that they are inter-locked processes, indivisible, and essential for optimum coding.

We conjecture that neurons have an internal decoder that is similar in form to a dynamic (adapting or moving) threshold (for example, see Buller et al., 1953; Hagiwara, 1954; Geisler and Goldberg, 1966; Brandman and Nelson, 2002; Chacron et al., 2003; Jolivet et al., 2004; Brette and Gerstner, 2005; Kobayashi et al., 2009; Fontaine et al., 2014). A dynamic threshold was originally proposed to explain spike-timing and used exclusively in forward (spike-generation) models. The dynamic threshold is so-called because it describes the time course of the neuron's firing threshold immediately following a spike. The canonical view that the spike threshold is constant (typically in the range of about −55 to −45 mV) fails to explain the increased refractoriness of a neuron, and hence the increase in time between spikes, in response to a constant stimulus (Hagiwara, 1954). In contrast, a dynamic (time-varying) threshold can capture at least some of the spike-timing features of real neurons.

Of importance to this work are two observations: (1) the time-dependent change in the threshold in response to the neuron's own spike can be viewed as an impulse response. The cumulative change in the threshold over the history of prior spiking activity is a summation of these individual impulse responses. That is, it is a convolution. This point is central to this work and provides a rationale for describing the threshold as a decoder. (2) Historically (since Buller et al., 1953) the threshold impulse response has been modeled as a decaying (or relaxing) potential, that is, it has low-pass characteristics. Most often, this decaying potential has been modeled as a single exponential of the form *h*(*t*) = *A* exp (−*t*/τ) (for example, see Chacron et al., 2003; Fontaine et al., 2014) or a mixture of exponentials of the form ∑_*i*_ *A*_*i*_ exp (−*t*/τ_*i*_) (for example, see Kobayashi et al., 2009). Based on these observations we will show that the time-varying threshold is a reconstruction filter, a decoder that can be optimized for energy-efficient and high-fidelity coding.

The idea of interpreting the above function *h*(*t*) as a decoding filter is motivated by post-synaptic filtering of spike trains. In this well-known motif^1^ (for example, see Hille, 2001), a neuron encodes an input *s*(*t*) in a spike train which is then filtered by a passive post-synaptic membrane that reconstructs *s*(*t*). This filter is usually modeled as a simple *RC* (resistance-capacitance) element specified by *A* exp (−*t*/τ) where τ is the membrane time constant (τ = *RC*) and *A* is a gain that governs the maximum size of the post-synaptic current in response to a single spike. The passive-membrane as a filter offers the simplest form of signal reconstruction or decoding, and shares similarity in form with the dynamic threshold [*h*(*t*), see above]. Thus, we conjecture that the dynamic threshold *h*(*t*) has been selected to mimic passive membrane dynamics. The cell membrane (lipid bilayer with leakage channels) is an ancient structure going back to the earliest single-celled organisms, and its low-pass filter characteristics are possibly just as old. Thus, an in-built decoding mechanism based on a blind evolutionary convergence to low-pass dynamics would confer selective advantage on the organism. Stated in other words, any internal information on the quality of coding is likely to exert favorable selective pressure. We argue that the neuron threshold provides exactly this information.

The threshold for spiking is an intrinsic component of any spiking neuron. It determines successive spike times and its value (in mV) is usually inferred from the measured action potential. How this value is set and whether the precise times of successive spikes are governed by an over-arching principle has received less attention, except insofar as to explain observed data (see Brette, 2015). Here, we provide an interpretation of the neuron threshold. We propose that the hypothetical post-synaptic membrane (decoder) is mirrored internally as a time-varying threshold *h*(*t*), and further, we show that it serves two purposes: (1) it regulates the long-term excitability of the neuron (the energy constraint), and (2) it provides an internal reconstruction *r*(*t*) by filtering the neuron's own spike train, and makes available the error signal *s*(*t*) − *r*(*t*) (coding fidelity). The neuron minimizes the error by firing a spike whenever the error exceeds a spiking threshold γ. Thus, our interpretation of the neuron threshold is that it is a bound on coding error.

We derive the optimal value of γ and show that it is signal-dependent, i.e., γ = γ (*s*, *t*), but reaches an asymptotic value in the limit of high signal amplitudes. This optimal firing threshold is derived for a piece-wise constant input signal, i.e., constant within an interspike interval, an approximation which holds in the limit of high spike-firing rates. Throughout this work we refer to the optimized process (the functions *h*(*t*) and γ (*s*, *t*)) as the optimal neural coder or simply as optimal coder.

While it is recognized that spike-times can be accurately predicted by neuron models with a dynamic threshold (see references above), our interest here is to show that optimum timing can be achieved with the proposed optimal coder. Spike-timing is simply an outcome that is testable. Thus, after establishing the optimized function γ (*s*, *t*), we test the coder with *in vivo* extracellular spike-timing data taken from primary electrosensory afferents of weakly electric fish and *in vitro* intracellular data taken from neocortical pyramidal neurons of the rat. There is a close match between predicted and experimental spike-times for these widely differing neurons. This provides evidence to support the hypothesis and suggests future lines of work. Finally, a time-varying threshold is a fairly old idea going back to Buller et al. (1953) and Hagiwara (1954) where it was first introduced mathematically to explain negative interspike interval correlations. More recently it has been recognized that the threshold can be signal-dependent (see Platkiewicz and Brette, 2010; Fontaine et al., 2014). What we provide here is a new perspective on the relevance of the neuron threshold to encoding and decoding. Our work suggests that a neuron is a highly precise coder, neither noisy nor unreliable as believed, but embodying a sophisticated coding system that can husband the neuron's energy resources judiciously without sacrificing coding fidelity. In this process, the neuron threshold plays a key role in regulating fidelity.

## Materials and methods

Experimental work on weakly electric fish was carried out at the University of Illinois at Urbana-Champaign, USA, with approval from the university IACUC. The pyramidal cell data set used here is from the rat, and was collected by Thomas Berger and Richard Naud in the laboratory of Henry Markram at École Polytechnique Federale de Lausanne (EPFL), Switzerland. The EPFL data is available in the public domain through the International Neuroinformatics Coordinating Facility (INCF) 2009 Spike Time Prediction Challenge^2^.

### Electrophysiology in weakly electric fish

Surgical and electrophysiological recording procedures in the weakly electric fish follow those reported in Nelson et al. (1997). The fish used in the study are of unknown sex. Briefly, adult brown ghost knife fish (*Apteronotus leptorhynchus*, 12–17 cm long), a species of gymnotiform fish, were lightly anesthetized by immersion in 100 ppm tricaine methane-sulfonate (MS-222, Sigma) for 2 min, and then immobilized with a 3 μl intramuscular injection of 10% gallamine triethiodide (Flaxedil, Sigma). The fish was restrained in a holding tank containing water and actively ventilated via a mouth tube. A surgical incision was made on the skin just posterior to the operculum to expose the posterior branch of the anterior lateral line nerve (pALLN). The nerve fiber from a P-type (probability coding) primary electrosensory afferent was isolated and its action potentials were recorded using glass micropipettes filled with 3 M KCl solution. Spike times and their associated spike waveforms were sampled and stored for offline analysis (at 60 μs resolution). The ongoing electric organ discharge (EOD) generated by the fish was monitored with a pair of carbon electrodes placed near the head and tail of the fish. Stimulation was provided by modulating the EOD with a single-cycle raised cosine of 100 ms duration and delivered across the whole body using two carbon rods placed on opposite sides of the fish along the anterior-posterior axis. The stimulus amplitude was calibrated with respect to the transdermal potential, measured between a recording electrode close to the skin on the lateral trunk of the fish and a reference electrode inserted under the skin on the dorsal surface. A 1 mV root mean-square (RMS) voltage increase was defined as the reference (0 dBV). Stimulus intensities ranged from 0 dBV to −60 dBV attenuation in 5 dB steps with 20 stimulus repetitions at each amplitude. Stimulu**s** waveforms time-locked to the neural data were stored for offline analysis.

### Rat pyramidal neurons (INCF data-set)

The 2009 INCF Spike Time Prediction Competition provides data-sets^3^ with a challenge to reproduce the spike times using a computational model (for results of the competition see Gerstner and Naud, 2009). Stimulus data are also provided although the competition did not require that the stimuli be reconstructed from the given spike activity. The data set from Challenge A (one of four challenges) is considered here, and is available in the public-domain along with a complete description of the methods from the organizers (http://www.incf.org/community/competitions/archive/spike-time-prediction/2009/challenge-a). Briefly, the data were obtained from *in vitro* current-clamp recordings in the soma of L5 pyramidal cells in the primary somatosensory cortex of the rat. The sex of the rat was not made known. The voltage data was filtered (2.4 kHz bandwidth Bessel filter) and sampled at 100 μs resolution. Recordings of somatic membrane potential were obtained in response to 60 s of injected current. The stimulus was repeated for a total of 13 trials. The stimulus and the first 39 s of the response from each trial was made public, whereas the remaining 21 s of the response remained private and was reserved for testing by the organizers of the competition. The stimulus consisted of the following sequence: (1) step current input with a duration of 2 s, repeated four times with an inter-stimulus duration of 2 s. The total duration was 17.5 s, (2) injection of 2 s of white noise, (3) six simulated spike trains generated by an inhomogeneous Poisson process convolved with exponential decays of different time constants and summed together for a duration of 42.5 s. The intensities were chosen randomly over 300–500 ms blocks to elicit firing rates between 5 and 10 Hz. From the voltage recordings, spike-times were found using a threshold of 0 mV. This study used the first 39 s of the publicly available current-clamp stimulus and the spike-times extracted from the 13 trials. The challenge required only the spike times to be predicted using a generative model of neural coding, but did not require the stimulus to be reconstructed.

### Optimum encoder formulation

**Figure 1:**
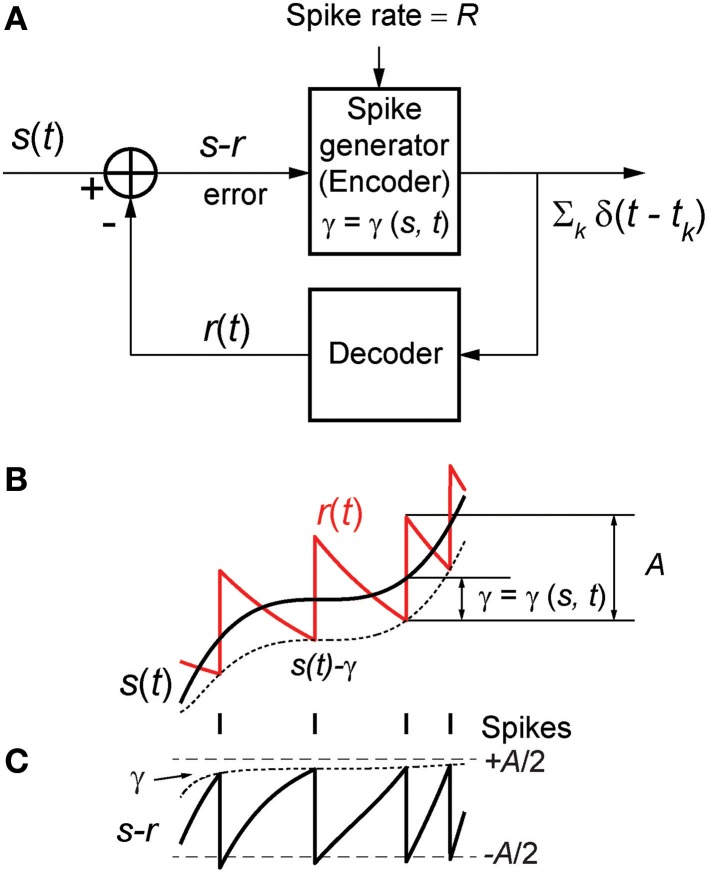
**(A)** The optimum neural coder consists of a spike generator (encoder), reconstruction filter (decoder), and a firing threshold (γ). Input to the neuron is *s*(*t*) > 0 and output is the spike train ∑_*k*_ δ(*t* − *t*_*k*_). The decoder reconstructs the signal *r*(*t*) from the spike train and provides an error feedback (*s* − *r*) to the spike generator. When the error reaches a level-dependent firing threshold γ = γ (*s*, *t*) the neuron outputs a spike. An optimization procedure (firing-rule), constrained by fixed energy (a fixed spike-rate *R*), determines the threshold for firing (see Mathematics and Equations). **(B)** Signal (black), its reconstruction (red), and threshold function for firing (dotted line). The reconstruction filter mimics a post-synaptic membrane, here a first-order low-pass filter (*Ae*^−*t*/τ^), and is a form of adapting or moving threshold. **(C)** The error signal *s* − *r* can be interpreted as another form of adapting threshold that causes the neuron to fire when the error reaches γ from below. The simplest firing rule is obtained in the asymptotic case *s*(*t*) ≫ *A*. In this case γ = *A*/2 and the optimal firing time occurs whenever *s* − *r* = *A*/2 (upper dashed line). The general form of the firing threshold is signal level-dependent (see Mathematics and Equations).

We consider here a neural coder that embodies a spike generator (encoder) and a reconstruction filter (decoder) *h*(*t*) within the same neuron (Figure 1A). The encoder portion of the neuron takes an input signal *s*(*t*) and generates a spike train representation ∑_*k*_ δ(*t* − *t*_*k*_) of the signal, where δ(*t*) is the Dirac delta and *t*_*k*_ are the spike-times indexed by *k*. The decoder portion of the neuron, with a reconstruction filter *h*(*t*), takes the spike train as input and generates a reconstruction *r*(*t*) of *s*(*t*) given by $r(t)=\sum_{k}\int_{0}^{t}\delta (\tau -t_{k})h(t-\tau )\;d\tau$. From Figure 1B it can be seen that the reconstruction has a form that is similar to a time-varying threshold used extensively in the literature for modeling threshold dynamics (see Introduction). The reconstruction error (coding fidelity) is *s*(*t*) − *r*(*t*). What is novel in the proposed coder is that this instantaneous error signal is available internally.

The motivation for the proposed coding/decoding mechanism comes from the classical pre- and post-synaptic pair of neurons (Abbott and Regehr, 2004; see also Hille, 2001). In this motif, a neuron encodes an input *s*(*t*) in a spike train which is then filtered by a post-synaptic membrane (decoder) to reconstruct *s*(*t*). The post-synaptic membrane is usually considered to be a simple first-order low-pass filter (an *RC* element) with exponential decay given by *A* exp (−*t*/τ), where τ = *RC* is the time-constant and *A* is the gain. In the proposed neural coder, the hypothetical post-synaptic membrane is mirrored in the neuron's internal decoder (the filter *h*(*t*)) as a time-varying threshold. We presume that this mechanism may have resulted from selective pressure so as to minimize coding error (see Introduction and Discussion for a detailed explanation).

Figure 1B depicts the optimal coding process for an arbitrary input *s*(*t*) (black trace) and reconstruction *r*(*t*) (red trace). The two processes, encoding and decoding, are coupled with the encoder emitting a spike only when the error *s*(*t*) − *r*(*t*) reaches a threshold γ (γ > 0). When a spike is initiated the neuron's threshold is instantly raised, and thereafter relaxes without resetting. The neuron will fire a spike at time t^*^ (Figure 1B) when the error satisfies

(1) $$s(t^{*})-r(t^{*})=\gamma .$$

The firing threshold is approached from above. In general the firing threshold is a signal-dependent function γ = γ(*s*, *t*). Thus, the firing threshold can be interpreted as the imposition of a bound on the permissible reconstruction error. It establishes the trade-off between energy consumption of the neuron and the coding fidelity. We conjecture that it is a biophysical analog of the error signal *s*(*t*) − *r*(*t*) (Figures 1B,C) that drives the spike generator in real neurons.

We propose that the encoding parameters should be chosen to minimize the squared reconstruction error $T^{-1}\int_{0}^{T}{\left(s(t)-r(t)\right)}^{2}dt$ subject to an energy constraint over the time window *T*. A simple first approximation of energy use (Levy and Baxter, 1996) is a linear function of the average spike-rate, *E* = *b* + *kR* where *R* is the average spike-rate in the window *T*, *k* is the cost per spike and *b* is a baseline energy consumption level. For a neuron with a given energy constraint, the constrained optimization problem with unknown γ and unknown filter parameters (*A*, τ), is

(2) $$\underset{\left(A,\tau ),\;\gamma =\gamma (s,t\right)}{Min}\left\{\;\int_{0}^{T}(s(t)-r(t){)}^{2}\;dt\;\right\},\text{ such that }E=\;b+kR.$$

An equivalent way of expressing Equation (2) is to state it in a Lagrangian form by introducing a Lagrange multiplier λ > 0 which weighs the constraint against the encoding error (Boyd and Vandenberghe, 2004). The Lagrangian form of the optimization problem is then (Boyd and Vandenberghe, 2004)

(3) $$\underset{\left(A,\tau ),\;\gamma =\gamma (s,t\right)}{Min}\left\{\int_{0}^{T}(s(t)-r(t){)}^{2}dt+\lambda (b+kR-E)\right\}$$

The formulations, Equations (2) and (3), are equivalent in the sense that for some value of the constraint *E* there exists some multiplier λ such that the solutions to Equations (2) and (3) are the same. The advantage of stating the optimization problem in the Lagrangian form is that it makes explicit that the terms λ*b* and λ*E* are constants and thus they do not affect the optimum value. The energy constraint is therefore equivalent to constraining the spike-rate *R*. The spike-rate *R* is a “design” parameter that is fixed prior to the optimization. Without the constraint, the optimization would drive the rate to the maximum possible firing rate of the neuron so as to drive the error to zero. When matching experimental spike trains, the rate *R* is computed from the observed long-term average spike output.

The outcome of the optimization process is the optimum firing threshold γ = γ (*s*, *t*) and the optimal reconstruction filter parameters (*A*, τ). The firing threshold provides the necessary trade-off between energy and coding fidelity (Figures 1B,C). Equations (2) or (3) specifies a functional relationship (the optimal trade-off) between spike-rate *R* and encoding error *E*(*R*) for the optimal coding neuron. We provide examples of *E*(*R*) later in the Results. The spike-rate constraint *R* determines the exact point along this curve where a given neuron will be located and hence, the coding fidelity. From computational or modeling perspective, when designing an optimal encoding neuron, a suitable (*A*, τ) pair is picked so that the rate constraint *R* is satisfied (as detailed further in Optimum Parameters for a First Order Reconstruction Filter). Thereafter γ (*s*, *t*) is determined, and an optimal spike-train can be generated. However, when working with experimental data, we are confronted with the “reverse” problem. We are given a set of spike-times, and we must determine whether these spike-times were generated according to the optimal encoding principle. We have to infer the parameters (*A*, τ) and γ (*s*, *t*) so as to predict, i.e., match, the known spike-times. This is shown in the following section.

#### Optimization procedure for comparison against experimental data

Our goal here is to verify the optimal coder predictions using experimental data. When working with experimental spike data, the only known quantities are the spike-times and the average spike-rate *R*, and we have to determine (*A*, τ), and γ (*s*, *t*) so that the spike-times and the rate *R* are matched. This is a multi-step procedure involving two separate optimizations [determine (*A*, τ) and then determine γ (*s*, *t*)] that jointly satisfy Equation (2) [or equivalently Equation (3)]. Both optimizations satisfy the constraint *R*, but the optimization of (*A*, τ) additionally provides the best match to the spike-times.

For illustration, consider the case where spike-trains are generated from *M* repetitions of a stimulus. Consider any one of the *M* spike-trains to be a reference trial. The reference trial is used to optimize the parameters. The optimal encoder is then validated by predicting the spike-times in the remaining (M−1) trials.

1. *Match spike-rate *R* by determining A and* τ: For the given spike-train with average spike-rate *R*, and a reconstruction filter *h*(*t*) = *A* exp (−*t*/τ), we can determine either *A* or τ, but not both (see Optimum Parameters for a First Order Reconstruction Filter). We pick a τ (from a set of τ s) and fix *A* so that the spike-rate constraint is met. Thus, (*A*, τ) are known.
2. *Minimize coding error*: Given a spike-rate *R* and the filter parameters (*A*, τ) from Step 1, we minimize the mean squared reconstruction error
  (4) $$\underset{\gamma =\gamma (s,t)}{Min}\left\{\int_{0}^{T}(s(t)-r(t){)}^{2}dt\right\},\text{ given }R,\text{ and }(A,\tau ).$$
  This yields the optimum firing policy or firing threshold γ = γ (*s*, *t*). The solution to the optimization problem is available in closed-form and is described in Optimum Value of γ for High Spike-rates with a Complete Derivation in Mathematics and Equations.
3. *Match spike-times*: A spike train is generated using the parameters determined in Steps 1 and 2. The spike-times are compared with spike-times from the reference trial, and a spike-time coincidence metric is determined (see Coincidence Measure for Similarity of Spike Trains).

Steps 1–3 are repeated over a range of τ values thereby generating a set of coincidence measures. The τ value with the highest coincidence is selected. Thus, the optimal (*A*, τ) is now known, and γ (*s*, *t*) can be determined. This set of parameters is considered to be the optimal parameter set, and is used to generate (*M* − 1) spike-trains. The average coincidence measure, averaged over all (*M* − 1) trials, is a measure of performance of the optimal coder. Section Parameter Selection provides additional details on the procedure for the p-type afferent and cortical neuron data.

#### Optimum parameters for a first order reconstruction filter

The simplest model of a post-synaptic membrane is a *RC* element (first-order low-pass filter) with impulse response given by

(5) h(t)=Aexp(−t/τ), t≥0.

The parameters τ and *A* define this reconstruction filter. The average output of the filter in response to a single spike is obtained by integrating Equation (5), and yields *A*τ/spike. We note that the average filter output should match the average level of the signal over a long time window, where fluctuations in the input signal are averaged out. Given an average spike-rate *R*, the average output level is *A*τ*R*. If the mean input signal level is *S*, then the filter parameters should be chosen to satisfy

(6) $$A\tau =\frac{\bar{S}}{R}.$$

While the right-hand side of Equation (6) is known from experimental data, the equation does not uniquely specify *A* and τ, and so this gives us one degree-of-freedom. This single degree-of-freedom is utilized to obtain the best match between the predicted and experimental spike-times (via the coincidence measure, described in Coincidence Measure for Similarity of Spike Trains). Thus, the optimal parameters (*A*, τ) are defined as those that satisfy Equation (6) and which provide the best coincidence with the experimental spike-times. We determine the optimal (*A*, τ) pair using a brute-force search over a range of τ values. For each value of τ, we obtain *A* from Equation (6). The (*A*, τ) parameters along with the spike-rate *R* are then used to generate a spike-train as detailed in Steps 2, 3, of Optimization Procedure for Comparison against Experimental Data.

#### Optimum value of γ for high spike-rates

For a positive signal *s*(*t*) > 0, given *R*, and (*A*, τ), Equation (4) will determine γ (*s*, *t*). In general this optimization problem is difficult to solve. However, in the limit of high spike-rates, it is possible to derive an expression for the optimal firing threshold. As the time between spikes decreases, the signal can be considered to be approximately constant between two spikes. As the spike-rate increases this piece-wise signal becomes an increasingly better approximation of the input signal. Given this assumption, we obtain a closed-form deterministic and causal firing-rule γ (*s*, *t*) that is dependent only on the dimensionless amplitude ratio ε = *s*(*t*)/*A* (see Section Mathematics and Equations for the development of the result),

(7) $$\gamma (s,t)=A\left\{\frac{(1+2\varepsilon )-\sqrt{1+4\varepsilon^{2}}}{2}\right\},\text{ where }\varepsilon =s(t)/A.$$

A spike is fired at the instant *s*(*t*) − *r*(*t*) = γ (*s*, *t*) (see Figures 1B,C). An asymptotic form of this firing rule when ε ≫ 1 (*s*(*t*) ≫ *A*, large signal) is particularly simple. The threshold for firing is constant and given by,

(8) γ=A/2, when s(t)≫A.

This result (Figure 1C, upper dashed line) holds for a wide range of signal amplitudes (see Mathematics and Equations). The more general time-varying firing threshold given by Equation (7) is shown as a dotted line in Figure 1C. This value of gamma defines a firing-rule which, in general, depends on time and the input signal level. The spike-generator models that are closest to the work described here are the dynamic-threshold models with non-resetting inputs (Brandman and Nelson, 2002; Kobayashi et al., 2009). In those models they assume that the firing-rule is γ = 0, so a spike is fired when *s*(*t*) − *r*(*t*) = 0. This rule will result in higher reconstruction errors compared to the optimal rule specified in Equations (7) or (8). Throughout this work, the optimal, signal-dependent form of γ given by Equation (7) is used to generate spike-trains.

### Leaky integrate-and-fire (LIF) neuron with dynamic threshold (LIF-DT)

The classical leaky integrate-and-fire or LIF neuron (Lapicque, 1907; Stein, 1967; for a brief history see Brunel and van Rossum, 2007) is a standard and widely used neuron model. In the original form it has a fixed threshold. Although it is well-known that the LIF model does not capture many of the spiking phenomenon observed in real neurons, it is commonly used, simple to implement, and is analytically tractable. It is defined by a differential equation, representing the membrane potential *V*(*t*), given by

(9) $$\tau_{m}\frac{dV(t)}{dt}=-V(t)+RI(t),$$

where *R* represents the membrane resistance, τ the model time-constant, and *I*(*t*) the input current. When the membrane potential *V*(*t*) exceeds a threshold θ, a spike is fired and the membrane potential is reset to zero. To encode the EOD waveform of the weakly electric fish, the waveform is first rectified before running the LIF model.

The classical LIF model can be extended in many ways, including incorporating a dynamic or adaptive threshold θ (*t*) (for example, see Chacron et al., 2001; Liu and Wang, 2001). Defining θ (*t*) = *h*(*t*) * ∑(δ(*t* − *t*_*k*_)) with *h*(*t*) = *A* exp (−*t*/τ) gives an exponentially decaying threshold. This model has two non-linearities: the spike-firing threshold and the resetting integrator (typically a hard reset to 0). Although similar to the proposed neural encoder, the dual non-linearities can lead to different behavior. We will compare the predictions of the optimal neural encoding model, the classical LIF model, and the LIF with Dynamic Threshold (LIF-DT) model.

### Coincidence measure for similarity of spike trains

The goodness of fit between the spike times recorded experimentally and the spike times generated by the optimal encoder was measured by the coincidence factor (Kistler et al., 1997). Experimental spikes and predicted model spikes are considered to be coincident if they occur within Δ seconds of each other. The coincidence factor is defined as,

(10) $$\Gamma =\frac{N_{\text{coin}}-E[N_{\text{coin}}]}{\left(N_{\text{data}}+N_{\text{model}}\right)}\frac{2}{\left(1-2\nu \Delta \right)},$$

where *N*_coin_ is the number of coincident spikes, *E*[*N*_coin_] is the expected number of coincidences for a homogeneous Poisson process with the same spike rate as the model, *N*_data_ is the number of experimental spikes, *N*_model_ is the number of model spikes, and ν is the spike rate of the model. This factor will reach 1 if every spike is coincident. The expected value of the coincidence factor is 0 if the model spike train is given by a Poisson process with the same rate as the experimental data. For this work, the coincidence window for the weakly-electric fish data was taken to be half an EOD period, as P-type afferents fire at most one spike per EOD period. For the INCF competition challenge data, the window was 4 ms.

### Reconstruction error

Reconstruction error was calculated from the RMS value of the error normalized by the RMS value of the stimulus, and reported as dBV RMS (re: stimulus)

(11) $$10\log_{10}\frac{{\left(\int_{0}^{T}(s(t)-r(t){)}^{2}\;dt\right)}^{1/2}}{{\left(\int_{0}^{T}s^{2}(t)\;dt\right)}^{1/2}}.$$

Normalizing the error to the stimulus allows reconstruction error to be compared across stimulus levels.

### Parameter selection

**Figure 2:**
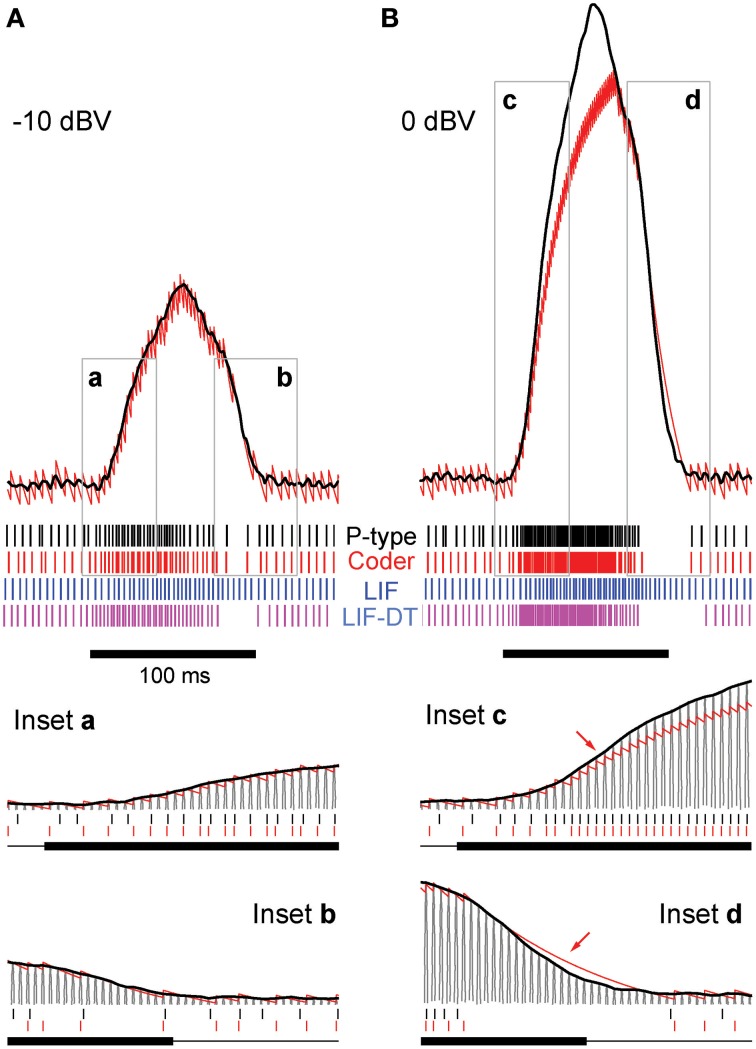
**Stimulus coding in an example P-type electroreceptor afferent, matched optimal neural encoder, leaky integrate-and-fire-neuron (LIF), and LIF with dynamic-threshold (LIF-DT) at two different stimulus intensities. (A)** −10 dBV, and **(B)** 0 dbV (drawn to scale). The stimulus as recorded at the skin of the fish (top, black trace) transiently modulates the ongoing electric organ discharge (EOD, removed for clarity) for 100 ms (black bar). The decoder output (optimal reconstruction) is overlaid (red trace). Spike trains are shown below the stimulus, P-type (black), optimum encoder (red), LIF (blue), LIF-DT (magenta). Reconstruction from LIF and LIF-DT neurons are not shown. Onset and offset periods (insets a-d) are magnified (lower panels) and show details of experimental vs. predicted spike-timing (coder only). The EOD (carrier) waveform is also shown with stimulus and reconstruction. Stimulus in **(B)** causes saturation in firing rate during the rise (arrow, inset c) and peak of the stimulus, and suppression of firing during the latter part of the decay (arrow, inset d).

**Figure 3:**
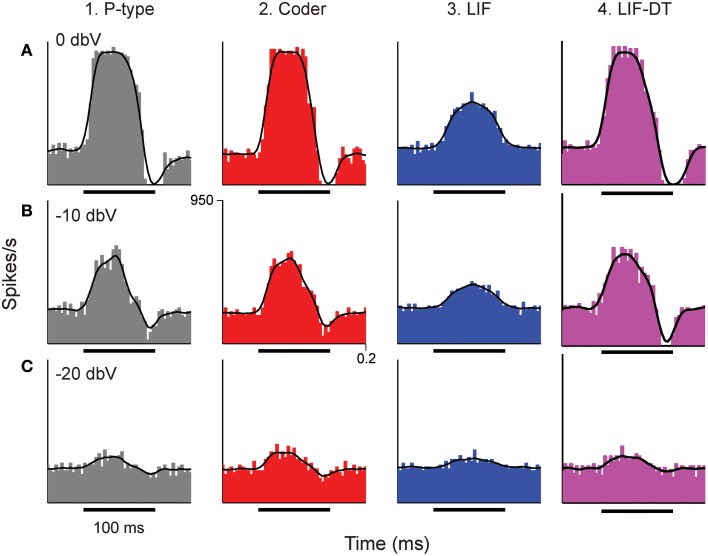
**Peri-stimulus time histograms (PSTHs) obtained in response to stimuli shown in Figure 2**. Columns from 1 to 4 are example P-type responses, spike-trains predicted by the optimal neural encoder, a LIF model, and a LIF-DT model, respectively. Rows show responses to stimuli of amplitudes 0 dBV **(A)**, −10 dBV **(B)**, and −20 dBV **(C)**. Single trials of the PSTHs shown in A and B are reported in Figures 2A,B, respectively. Each PSTH was determined from 20 trials (4 ms bins). Black traces are the smoothed histograms (16 ms window). The PSTH of the spikes simulated by the optimal encoder closely approximate the time-varying spike-rate seen in the P-type responses.

**Figure 4:**
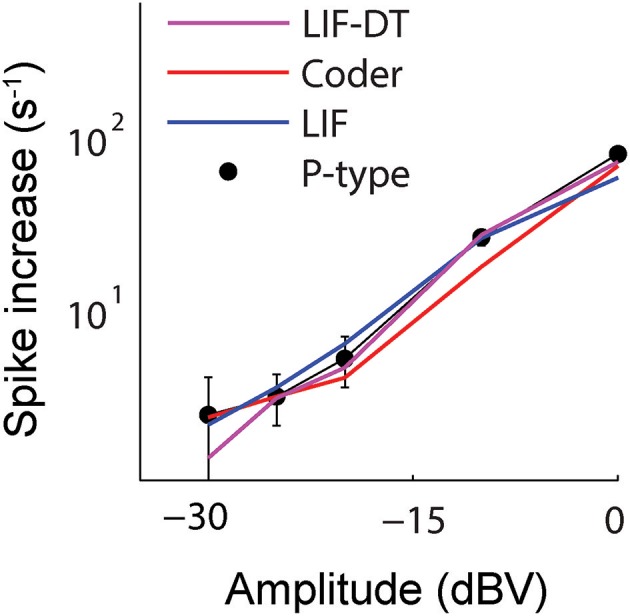
**Change in spike rate (ordinate) as a function of stimulus intensity (abscissa) for the P-type afferent (filled black circles) and matched optimal encoder (red), LIF model (blue), and LIF-DT model (magenta)**. The change in spike rate is calculated as the difference between the average spike rate elicited by the stimulus and the baseline, averaged over 20 trials. The increases in spike counts are the same in all three cases, thus suggesting a rate code. However, the P-type afferent and the LIF neuron do not have similar temporal spiking patterns (see Figures 2, 3).

**Figure 5:**
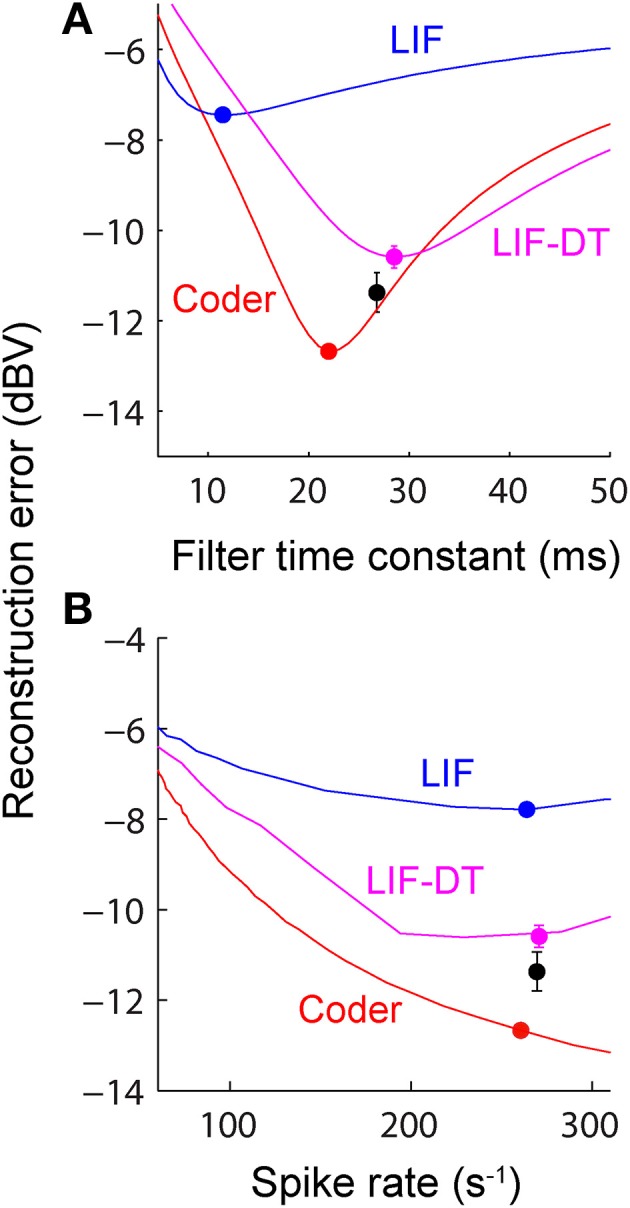
**(A)** Reconstruction error as a function of filter time-constant given an energy constraint (fixed spike rate). Experimental data are from a P-type afferent. For a given baseline spike rate (237 spikes/s, P-type, Figure 3) the reconstruction error is a function of the filter time-constant (red: encoder, blue: LIF, magenta: LIF-DT). The curve also depicts sensitivity of reconstruction error to fluctuations in the filter time-constant. For the reconstructions (shown later) we picked filter time-constants in the following way: The encoder has a built-in decoder which by optimization has a time-constant that gives the lowest reconstruction error (filled red circle, 22 ms). On the other hand, for the LIF neuron there is no built-in decoder but we picked a hypothetical decoder that would provide the lowest error (filed blue circle, 11.5 ms). Similarly for the P-type unit and LIF-DT model we estimated the best time-constant assuming a hypothetical decoder (black and magenta circles, 26.8 and 28.5 ms). See text for more details. **(B)** Reconstruction error as a function of spike-rate for the best filter time-constant (as shown in **A**). Symbols and lines are as in **(A)**. The lines represent simulations over the entire range of spike-rates shown in the abscissa. The filled circles mark the reconstruction errors at the experimentally determined P-type baseline spike rate (237 spikes/s), with a tolerance of ± 15 spikes/s due to simulation constraints. Broadly, the error should reduce with increasing spike rate due to improved signal representation. This is analogous to the rate distortion function (Cover and Thomas, 2006). As predicted the optimal encoder yields the lowest error for a given spike rate.

**Figure 6:**
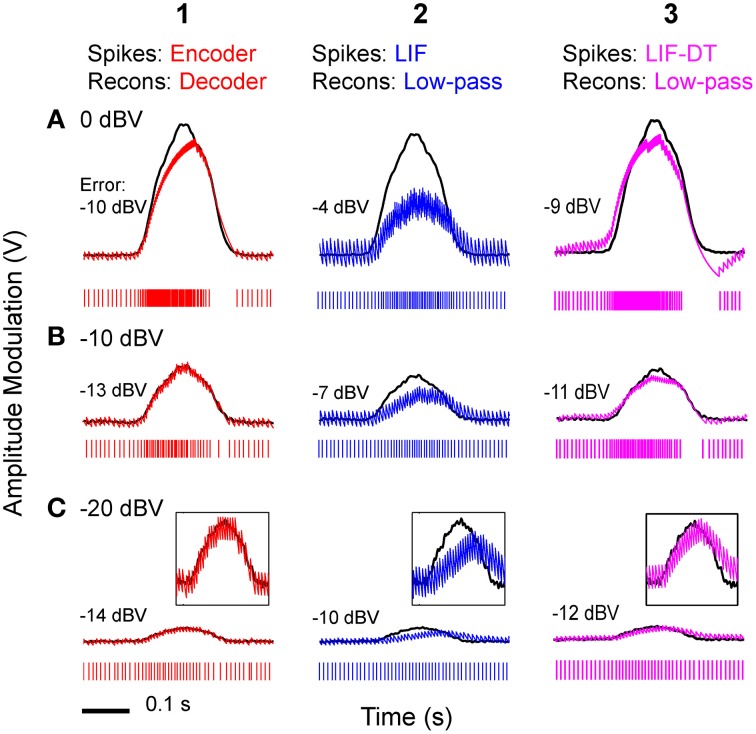
**Stimulus reconstructions from: (1) optimal coder, (2) LIF, and (3) LIF-DT spike trains using a low-pass filter at three stimulus levels. (A)** 0 dBV, **(B)** −10 dBV, **(C)** −20 dBV. Experimental data are from a P-type afferent. Each panel depicts the stimulus (black trace), and reconstructed stimulus (encoder: red, LIF: blue, LIF-DT: magenta) from one of 20 trials (spike train is shown below the stimulus). In Row **(C)**, insets provide magnified view of the stimulus and reconstruction. Reconstruction error in dBV (re: stimulus) is reported in each panel, with more negative values indicating smaller error. Reconstruction filters had time-constants that yielded the lowest reconstruction error (see Figure 5). The optimal decoder reconstructions track the stimulus onset and offset without noticeable delays, and match the stimulus amplitude. However, the LIF reconstructions suffer phase and amplitude distortion. The quality of LIF-DT reconstructions lie between those of the optimal decoder and the LIF neuron, with noticeable distortion at the highest signal amplitudes.

**Figure 7:**
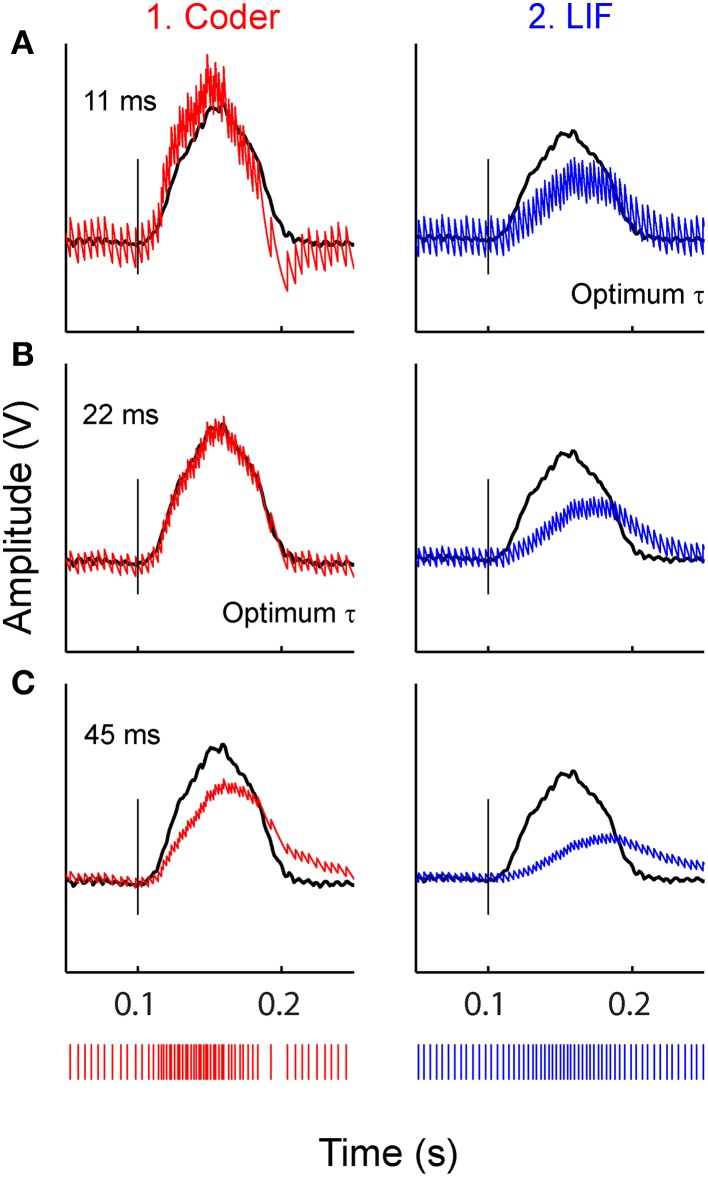
**Stimulus reconstructions from: (1) optimal coder (red) (2) LIF (blue) spike trains (bottom traces) using a low-pass filter with three different time-constants. (A)** 11 ms (optimum for LIF), **(B)** 22 ms (optimum for coder), **(C)** 45 ms (arbitrary value). Experimental data are from a P-type afferent. Stimulus is shown as black trace. Vertical line marks stimulus onset time. The optimum coder **(B1)** preserves onset time and stimulus features without significant distortions. At small time-constants **(A1)** the coder is sub-optimal and exhibits a faster rise-time and decay with rebound (re: stimulus) resembling the smoothed PSTH (see Figures 3, B2) but still tracks the onset accurately. At long time constants the coder exhibits phase delays and amplitude distortion. The LIF reconstructions (right column) demonstrate shifts in phase, amplitude, and smearing (lengthening) of the reconstruction (re: stimulus) even when the time constant is optimum **(A2)**. These figures suggest that the time-pattern of spikes are important for preserving temporal features of the reconstructed stimulus.

For the P-type spike trains, the stimulus (modulated EOD waveform) was recorded at the skin of the fish. It was filtered with a bandpass filter centered at the EOD frequency with a bandwidth of approximately 50 Hz. This preserved the EOD waveform and amplitude modulations while eliminating artifacts in the recorded stimulus. Baseline stimulus levels and firing rates, for use on the right-hand-side of Equation (6), were obtained from the pre-stimulus portion of the data. Due to the variability in estimating the baseline level and rate, (*A*, τ) pairs which resulted in simulated spike-trains within 15 spikes per second of the experimental data were allowed. Stimuli were measured at the skin of the fish with respect to a subdermal reference electrode placed on the dorsal side of the fish. These are most likely not the true potential difference across the receptor. They were rescaled with respect to the baseline EOD to correct for stimulus discrepancies. This affects the absolute value of the stimulus peak amplitude but preserves the differences between the stimuli (in dBV). For the optimal coder the scale factor was 4.95 (stimulus level: −20 dBV), 6.28 (stimulus level: −10 dBV) and 4.55 (stimulus level: 0 dBV). Where applicable, the parameter values used to generate Figures 2–**7** were τ = 34.5 ms and *A* = 2.05 × 10^−4^ V (−20 dBV), τ = 22 ms and *A* = 3.42 × 10^−4^ V (−10 dBV), and τ = 24.5 ms and *A* = 3.22 × 10^−4^ V (0 dBV). For each stimulus level, the parameters were chosen to maximize coincidence with the P-type spike-train from one randomly selected trial (out of twenty). The same parameters were used to generate spike-times for the remaining 19 trials. The parameters are similar across stimulus strength, with the small differences resulting from the small changes in the average baseline firing rate as measured in the pre- and post-stimulus periods.

The LIF model was tuned using the threshold parameter and the time-constant of the input filter. For simplicity, the input filter resistance (*R*) in Equation (9) was fixed at *R* = 1. The threshold level could then be adjusted to achieve the desired firing rate. Because of the variability in estimating the baseline level and spike-rate, parameter values which resulted in simulated spike-rates within 15 spikes per second of the experimental rate were allowed. The half-wave rectified EOD waveform was used as input to the neuron with EOD scale factor of 5.1 (−20 dBV), 4.9 (−10 dBV), and 4.2 (0 dBV). Where applicable, the parameter values used to generate LIF spike-trains and PSTHs shown in Figures 2–4 were τ_*m*_ = 50 ms and θ = 3.28 × 10^−5^ V (−20 dBV) τ_*m*_ = 114 ms and θ = 1.44 × 10^−5^ V (−10 dBV), and τ_*m*_ = 98 ms and θ = 1.62 × 10^−5^ V (0 dBV). As with the optimal coder, the LIF parameters were selected to maximize coincidence with the P-type spike-train from one randomly selected trial (out of twenty). The same parameters were used to generate spike times for the remaining 19 trials. The LIF and LIF-DT models do not have an in-built decoder. To generate decoded waveforms, it is necessary to choose a reconstruction filter for both these models.

To compare the LIF model with the optimal encoder, a first-order reconstruction filter was used to reconstruct the signal from the LIF spike-trains. The filter parameters were chosen to minimize the average reconstruction error between the EOD envelope and the LIF spike-trains. The optimum reconstruction filter parameters were determined through simulations, and the reconstructions depicted in Figures 5–**7** used the following parameters: τ = 27 ms and *A* = 2.58 × 10^−4^ V (−20 dBV), τ = 11.5 ms and *A* = 6.01 × 10^−4^ V (−10 dBV), and τ = 6.5 ms and *A* = 1.1 × 10^−3^ V (0 dBV). P-type afferents can fire a maximum of one spike per EOD cycle and so the optimal encoder and the LIF neuron were limited to firing at most once per EOD cycle.

To tune the LIF-DT model, a similar procedure to the LIF model was used. The input filter resistance was again assumed to be 1. The membrane time constant τ_*m*_, adaptation time constant τ, and adaptation height *A* were adjusted to achieve the baseline firing rate and maximize the coincidence with the P-type spike-train from a random trial. Due to the variability in estimating the baseline spike rate, parameter values resulting in a spike-rate within 15 spikes per second of the experimental rate were allowed. The same EOD scale factors were used as for the proposed neural encoding model. The half-wave rectified EOD waveform was used as input. The optimal parameters were *A* = 7 × 10^−5^ V, τ = 35 ms, τ_*m*_ = 2 ms (−20 dBV), *A* = 6.4 × 10^−5^ V, τ = 44 ms, τ_*m*_ = 1 ms (−10 dBV), and *A* = 6.4× 10^−5^ V, τ = 44 ms, and τ_*m*_ = 1 ms (0 dBV). Like the LIF model, the LIF-DT model has no notion of a decoded waveform. We therefore had to determine a low-pass reconstruction filter which minimized the average reconstruction error for the LIF-DT spike-trains. The reconstruction parameters were *A* = 2.1× 10^−5^ V and τ = 36 ms (−20 dBV), *A* = 2.6× 10^−4^ V and τ = 28.5 ms (−10 dBV), and *A* = 3.9× 10^−4^ V and τ = 20 ms (0 dBV). The LIF-DT model was constrained to fire at most one spike per EOD cycle.

**Figure 8:**
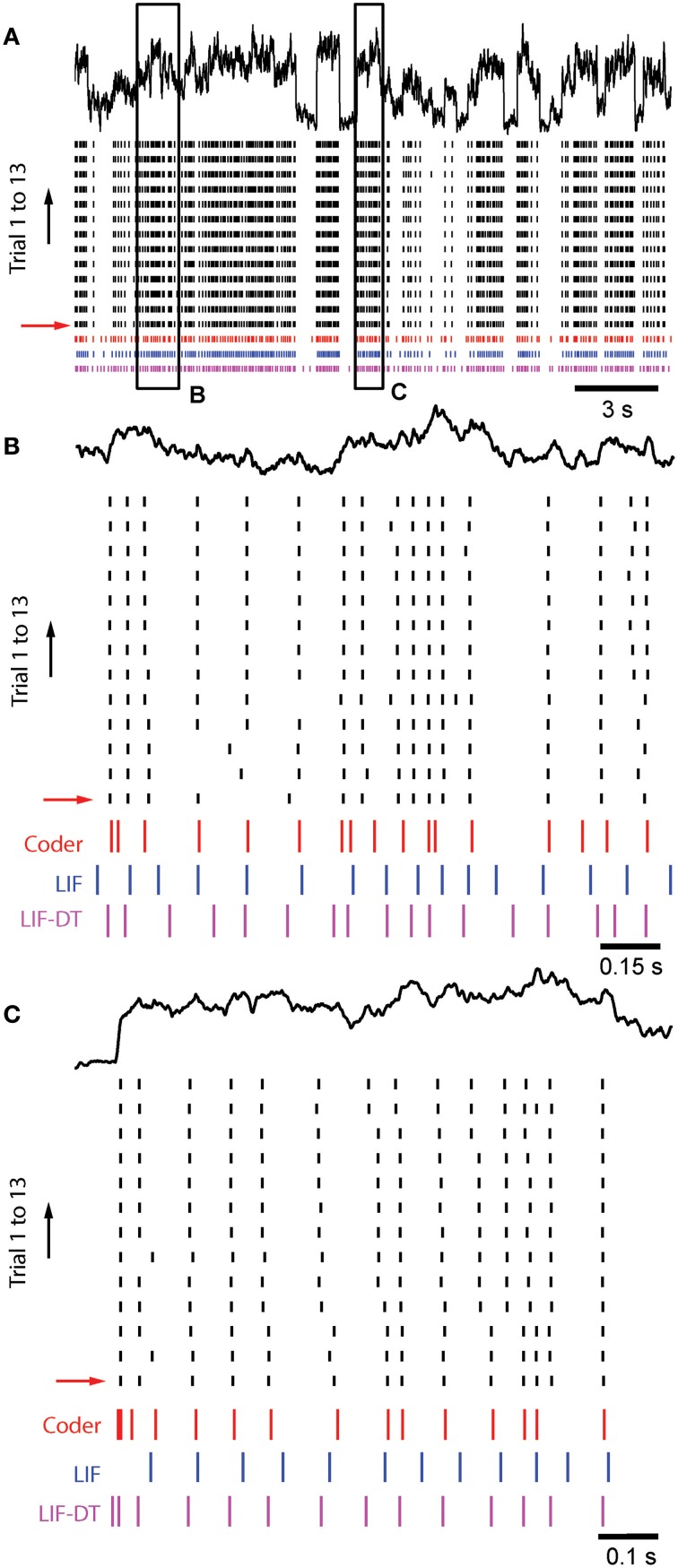
**Comparison of spike outputs from a cortical pyramidal neuron, optimal coder, LIF-DT and LIF neuron in response to frozen noise**. Data provided by the INCF Spike Time Prediction Challenge. **(A)** Stimulus trace (black), raster plots of cortical response to 13 trials (black), predicted coder spike train (red), predicted LIF spike train (blue), and predicted LIF-DT spike-train (magenta) over the entire duration of 21.5 s. Parameters for the optimal coder were matched to trial #1 (red arrow). The coder and LIF models' spike-times are deterministic and only one trial can be generated for each. **(B,C)** Expanded views of input and spike times for insets **(B,C)** (arbitrarily selected) shown in **(A)**. The optimal coder makes predictions of spike-times that are in good but not complete agreement with the experimental data. In general, coincidence with coder spike-times is high when the inter-trial variability in the experimentally determined spike-times is small. The LIF-DT model has almost equal coincidence. The LIF spike-times have poor coincidence.

**Figure 9:**
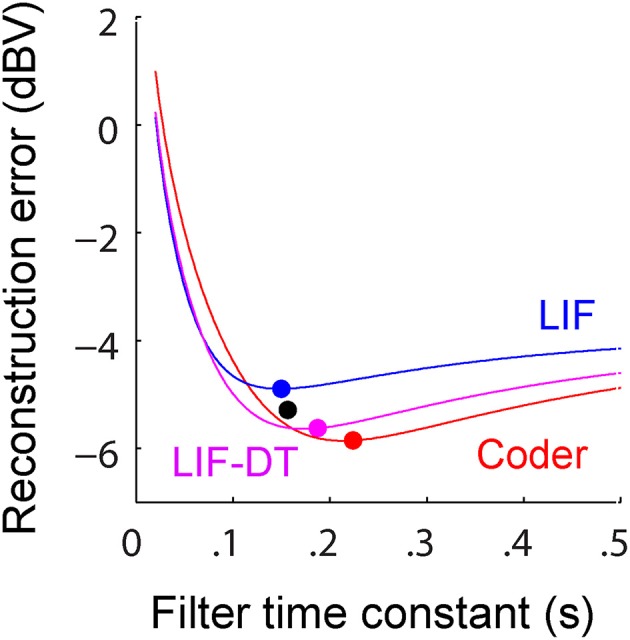
**Reconstruction error as a function of filter time-constant given an energy constraint (fixed spike rate)**. Experimental data are from cortical pyramidal neurons. For a given overall spike rate (9.74 spikes/s, cortical neuron, see Figure 8) the reconstruction error is a function of the filter time-constant. Explanation follows Figure 5. Best reconstruction filter time-constants are: 224 ms (encoder, filled red circle), 150 ms (LIF, filled blue circle), 187 ms (LIF-DT, filled magenta circle), and 157 ms (cortical neuron, filled black circle). Overall the optimal coder, and LIF and LIF-DT spike trains have similar coding fidelity even though their spike-timings are very different (Figure 8). See text for more details.

**Figure 10:**
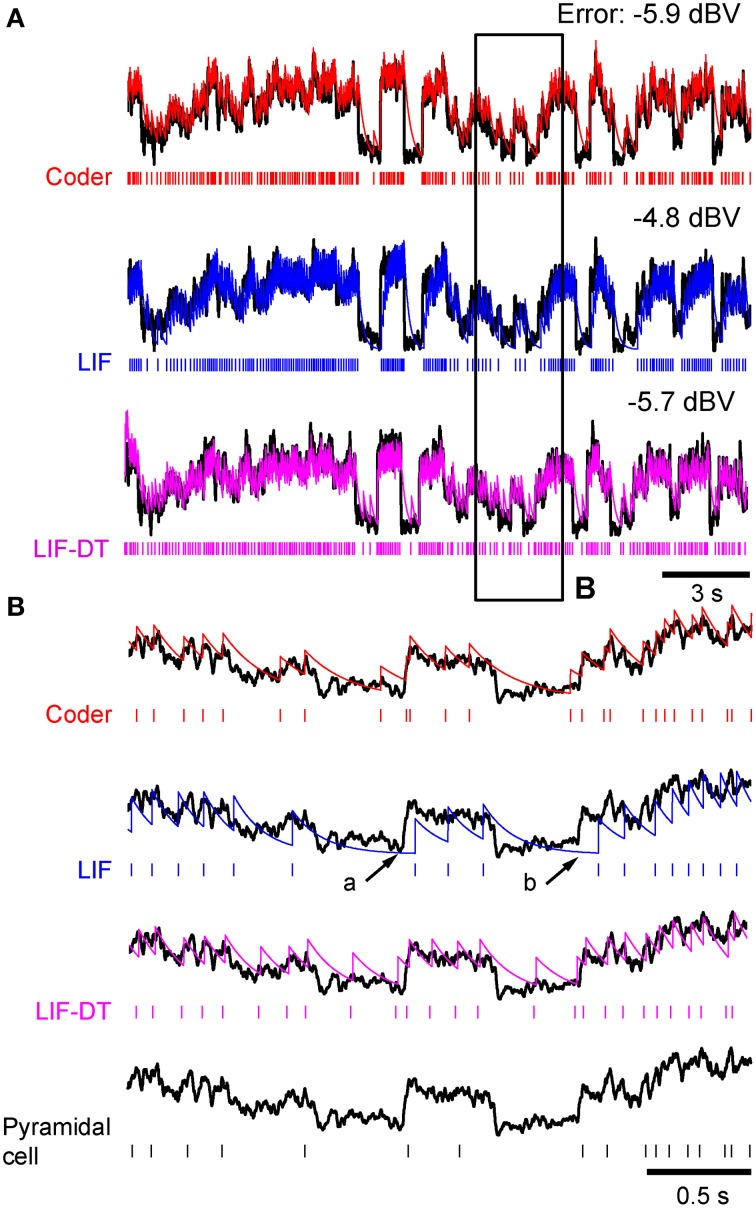
**Stimulus reconstructions from: (1) optimal coder (2) LIF, and (3) LIF-DT spike trains using a low-pass filter**. Experimental data are from cortical pyramidal neuron depicted in Figures 8, 9. **(A)** Stimulus (black trace), and reconstructed stimulus from encoder (top, red), LIF neuron (middle, blue), and LIF-DT neuron (bottom, magenta). Spike trains as shown in Figure 8A. Reconstruction error in dBV (re: stimulus) are reported for each trace, with more negative values indicating smaller error. Reconstruction filters had time-constants that yielded the lowest reconstruction error (see Figure 9). **(B)** Detail of inset shown in A provides magnified view of the stimulus and reconstructions. For comparison, stimulus and cortical neuron spikes are shown below the LIF traces. The optimal decoder reconstructions track the stimulus onset and offset without noticeable delays, and match the stimulus amplitude. However, the LIF reconstructions are delayed (e.g., arrows at a and b).

To fit the spike-times from the INCF competition (**Figures 8**–**10**), the current waveform was first filtered with a first-order low-pass filter with a gain of 10^6^ and a time-constant of τ_*m*_. The filter gain served to convert the input signal from hundreds of pA into the mV range. The three methods were used to predict the precise spike-times for one trial with parameters being adjusted to match spike times with highest coincidence factor Γ, Equation (10). For the neural source encoder, these parameters were: *A* = 1.5 mV, τ = 224 ms, and τ_*m*_ = 20.3. For the LIF model, the parameters were *R* = 1, τ_*m*_ = 225 ms and θ = 1.24 mV. The parameters minimizing reconstruction error for the LIF model were *A* = 2.2 mV and τ = 150 ms. For the LIF-DT model, τ_*m*_ = 13 ms, τ = 165 ms, and *A* = 2.7 mV. The parameters minimizing reconstruction error for the LIF-DT model were *A* = 1.6 mV and τ = 187 ms. For all three methods, parameter values which resulted in simulated spike-rates within 1.5 spikes per second of the experimental rate were allowed.

Reconstructions of the experimental spike-trains were created by finding the parameters *A* and τ which minimized the reconstruction error when the spike-trains were convolved with a filter *h*(*t*) = *A* exp (−*t*/τ). For the weakly electric fish data-set, the optimal parameters were *A* = 0.17 mV and τ = 45 ms (−20 dBV), *A* = 0.29 mV and τ = 26.8 ms (−10 dBV), and *A* = 0.4 mV and τ = 20 ms (0 dBV). For the INCF data-set, the parameters which minimized reconstruction error for the experimental spike-train were *A* = 2 mV and τ = 157 mV.

## Results

The optimal coder can be readily validated because its outcomes are a set of spike-times predicted by Equation (1). Further, for a given spike-rate (energy constraint), the internal decoder provides a reconstruction which has a higher fidelity (lower error) than other reconstructions. These predictions will be compared to spike-timing data obtained from peripheral sensory neurons in the electrosensory system of weakly electric fish (*in vivo*) and from cortical pyramidal neurons in the somatosensory system of the rat (*in vitro*). They will also be compared to spike trains predicted by a classical leaky-integrate and fire (LIF) neuron and a LIF neuron with a dynamic threshold (LIF-DT).

### Coding in primary electrosensory neurons of the weakly electric fish

Broadly, the key finding is that for a given constraint on the spike rate, the spikes are timed so that the decoded signal has minimum reconstruction error. The spike generator has access to the error signal, which allows spikes to be generated to minimize reconstruction error (Figure 1A).

#### Encoding and spike-timing

Figures 2A,B show the stimulus (black trace, A: −10 dBV and B: 0 dBV intensities) and spike response (black spikes) of a primary P-type electrosensory afferent from the weakly electric fish *Apteronotus*, the optimum reconstruction (red trace) and spike response (red spikes) of the neural coder, the response of a leaky-integrate-and fire (LIF) neuron (blue spikes), and the response of an LIF-DT neuron (magenta spikes). The energy constraint of the optimal coder was the baseline spike rate of the afferent (237 spikes/s), from which the filter parameters *A* and τ were determined using Equation (6). For each of 20 trials, the initial condition of the optimal decoder was set so that the time to first-spike of the encoder was the same as the time to first-spike of the P-type unit.

Starting with this initial condition, the decoder attempts to track the stimulus until the error *s*(*t*) − *r*(*t*) reaches the firing threshold γ (*s*, *t*) given by Equation (22) whereupon the encoder outputs a spike (see Figure 1B). Proceeding in this way, the neural encoder generates a deterministic spike train (Figures 2A,B, red spikes) where the spikes are timed at the discrete jumps in the reconstruction (red trace). Under baseline conditions, when there is no change in the input to the optimal coder, the spikes are produced at the rate set by the energy constraint. Despite the simplicity of the firing rule and the linear reconstruction, the optimal coder (Figure 1) differs from current spike generator models by using feedback to regulate reconstruction error.

The LIF and LIF-DT neurons were similarly tuned to match the first-spike time and thereafter generated spikes according to the dynamics prescribed by Equation (9) (the LIF-DT neuron additionally incorporated a dynamic threshold). There is no internal decoder or feedback in the case of the LIF and LIF-DT neurons, and so spikes were generated in a feed-forward manner. The firing threshold is a constant, and was determined from a match to the baseline firing rate.

At this amplitude (Figure 2A, −10 dBV) the peak firing rate is well below saturation, and there is good agreement with the experimental spike-times as seen from a comparison of the P-type and coder spike trains (top). Figure 2A insets magnify the time period around the stimulus onset (inset a) and offset (inset b). At the onset of the stimulus, as intensity increases, the optimal coder predicts that the neuron will rapidly fire spikes to encode the changing stimulus level with minimal error. At a mechanistic level this is due to a rapid build-up in the error *s*(*t*) − *r*(*t*) resulting in a shortening of the recovery period of the decoder following a spike (red trace). See also the amplitude-dependency of the decoder recovery in Figure 1C. Thus, more spikes will be output in a unit of time. The precision of spike-timing (re: experimental spike times) improves (see timing of last few spikes in inset a, vs. the first few spikes following stimulus onset). When the stimulus decays (inset b), the error is sub-threshold (*s*(*t*) − *r*(*t*) < γ), and the decoder goes into a free decay between spikes thereby lengthening the inter-spike interval or ISI. This is seen more clearly in inset b where the ISI between spikes #3 and #4 is longer for both coder (red spikes) and experimental P-type spikes (black spikes). Whenever the error grows slowly, the time for the error to reach the threshold will be longer, and so the time interval between spikes increases.

Stimuli that have high attack and decay rates and which drive a neuron into saturation (peak firing rate) and suppression (quench firing) serve to illustrate other aspects of the optimal coder (Figure 2B, 0 dBV). Following onset, the stimulus grows so rapidly that the decoder is unable to reduce the reconstruction error. Spikes are therefore fired at the maximal rate in an attempt to “catch-up” with the stimulus, reaching saturation well before the peak of the stimulus (inset c, red arrow). Thereafter, the source encoder and the afferent both fire at their peak rate. The reconstruction falls just short of the stimulus at the peak (red trace in Figure 2B).

On the decaying slope of the stimulus (inset d) the stimulus falls off rapidly, and its rate of decay is faster than the decay rate of the filter impulse response (inset d, time-interval around red arrow). At this point, the neural coder (and the afferent) cease to fire. It is illustrative to compare inset a with c, and inset b with d. The most accurate way to represent an extremely sharp onset (high attack rate) is to fire at maximal rates. Conversely, the most accurate way to represent a rapid offset (high decay rate) is to completely cease firing. This is commonly observed in many neurons that maintain a baseline or spontaneous discharge rate, for e.g., in auditory nerve fibers (Kiang et al., 1965) where the behavior is often described as “primary-like” response. Thus, the source encoder provides an explanation for the rapid changes in discharge timing at the onset and offset of stimuli, namely, spikes are placed only where they are needed. This is a major consequence of the energy constraint and a feature of optimal coding.

The deterministic optimal coder predicts the number of spikes and timing fairly accurately. The coincidence factor Γ (Kistler et al., 1997) averaged over 20 trials were 0.2 ± 0.06 (−10 dBV) and 0.49 ± 0.04 (0 dBV). These are conservative estimates because we used a small window of one-half the EOD period (0.6 ms). Any spikes falling outside this window were not considered to be coincident. While the features in the timing of P-type spikes are captured by the optimal coder, the LIF neuron encodes stimulus amplitude in its firing rate and does not predict timing (see spike output in Figures 2A,B). The coincidence values of 0.12 ± 0.04 (−10 dBV) and 0.19 ± 0.04 (0 dBV) are smaller than coincidence values for the optimal coder. The LIF neuron does not reproduce the suppression in firing observed in the afferent and encoder spike trains. Further, LIF firing exhibits a lag in following the stimulus. This can be seen most clearly at the stimulus onset (Figures 2A,B) and is discussed in detail later. The LIF-DT model has a higher coincidence factor than the LIF model, 0.16 ± 0.09 (−10 dBV), 0.42 ± 0.05 (0 dBV). Using a Mann–Whitney *U*-test for the 0 dBV stimuli, we reject the null hypothesis that the mean coincidences are the same between the deterministic optimal coder and LIF-DT model (*p* < 0.01). For the LIF-DT model and optimal encoder at the −10 dBV stimulation level, the difference in mean coincidence was not found to be significant. The mean coincidence for the optimal coder is significantly higher than the mean coincidence for the LIF neuron at both the 0 dBV and −10 dBV stimulus levels (*p* < 0.01).

The temporal features observed in the P-type, optimal coder, LIF, and LIF-DT neurons are reflected in the peri-stimulus time histograms (PSTHs, Figure 3) which report spike data over 20 trials at three intensities: Figure 3A (0 dBV), 3B (−10 dBV), and 3C (−20 dBV) for P-type afferent (gray, column 1), optimal coder (red, column 2), LIF neuron (blue, column 3), and LIF-DT neuron (magenta, column 4). The smoothed histogram is also shown as the black trace. The P-type afferent, optimal coder, and LIF-DT neurons have similar PSTHs and demonstrate response saturation (Figures 3A1,A2) and varying degrees of response suppression at stimulus offset (all intensities). It should be noted that the P-type PSTHs and the optimal coder PSTHs do not follow the stimulus (depicted in Figure 2). The LIF PSTH (column 3) on the other hand closely follows the stimulus but does not demonstrate response saturation or suppression. Broadly, a comparison of the optimal coder PSTHs (column 2) with experimental data (column 1) supports the assertion that the optimal coding principle captures spike-timing features accurately.

Aggregate spike-rates as a function of stimulus intensity were estimated for the spike-trains. Figure 4 shows the rate-intensity curve in logarithmic coordinates. Spike rate (ordinate) is depicted as an increase in average firing rate within the stimulus window over the baseline discharge rate. The P-type firing rates are obtained from experimental data and shown as points (filled black circles). The spike rates are in good agreement with one another over a wide range of stimulus amplitudes. All four neurons convey information about signal amplitude in the firing rate in nearly identical ways, even though LIF neuron spike-timing is in disagreement with the P-type afferent, the optimal coder, and the LIF-DT neuron (Figures 2, 3).

#### Decoding and reconstruction error

A major feature of the optimal coder is that it has an internal decoder that mirrors a hypothetical post-synaptic membrane, and this decoder is optimized to minimize the reconstruction error. In this work we consider a simple *RC* element (a first-order low-pass filter) as the decoder, although other types and orders of filters can be used as well. The first-order filter allows some simplifications and a closed-form solution of the optimal firing threshold (γ) (see Materials and Methods, and Mathematics and Equations). Our assertion is that the role of the neural firing threshold is to time spikes so that the reconstruction error is minimized. Figure 5A depicts the reconstruction error as a function of time constant when an energy constraint (spike rate) is imposed. For the P-type afferent shown earlier (Figures 2–4), the optimal coder (red trace) achieves minimum error when the decoder time constant is τ = 22 ms (filled red circle) with error of −12.7 dBV (standard deviation of 0.1). The trace was generated using spike data in response to −10 dBV. This time constant is in the same range as the mean time-constant of 16 ms reported for the post-synaptic neuron in the electric fish hindbrain (Berman and Maler, 1998).

The time constant obtained empirically via Equation (6) is close to this value across stimulus intensities with some variability (24.5 ms at 0 dBV, 22 ms at −10 dBV, and 34.5 ms at −20 dBV). The variability is due to small fluctuations in the inter-trial baseline firing rates, and is also a practical limitation of the use of an iterative approach to estimate the time constants from Equation (6). Broadly, these time constants are optimum or close to optimum for the given spike rate constraint, and will provide minimum-error reconstructions.

In contrast, the LIF and LIF-DT neurons are spike generators and do not have an internal decoder. However, we can assume that these model neurons synapse onto a first-order low-pass membrane solely for the purpose of signal reconstruction. For a given spike-rate the reconstruction error will be a function of time-constant. Figure 5A shows the reconstruction for the LIF neuron using spike data in response to −10 dBV. The optimum time constant is 11.5 ms (filled blue circle) with error of −7.4 ± 0.01 dBV. For the LIF-DT neuron the minimum reconstruction error was −10.6 ± 0.2 dBV. For comparison, we determined the time-constant for minimum error reconstruction using the experimentally determined P-type spike train in the same way (Figure 5A, filled black circle). The best time-constant is 26.8 ms, with error of −11.4 ± 0.4 dBV. Thus, the hypothetical decoder parameters and error for the P-type spike train are closer to those of the optimal decoder and LIF-DT neuron, but not the LIF. Broadly, Figure 5A supports the assertion that the reconstruction error is minimized during optimal coding. The optimal coder had significantly lower reconstruction error than the reconstructions from the LIF, LIF-DT, and experimental spike-trains (using a Mann–Whitney *U*-test with *p* < 0.01). These results should be interpreted with some caution, because they apply only to causal reconstructions with a first-order low-pass filter. Other types of reconstruction filters or reconstruction strategies, such as the use of a Bayesian estimator (Gielen et al., 1988) or a non-causal Wiener filter (de Ruyter van Steveninck and Bialek, 1988; Bialek et al., 1991; Gabbiani, 1996; Gabbiani and Koch, 1996) can lead to lower reconstruction errors. We show this later with reconstructions from a non-causal Wiener filter using the experimental spike-times.

Using the optimum time constants from the analyses shown in Figure 5A, we carried out reconstructions of stimuli from optimal coder, LIF neuron, and LIF-DT spike trains. Figure 6 depicts reconstructions for the spike trains at three different stimulus intensities (rows A–C), for optimal coder generated spike trains (red, column 1) LIF neuron spike trains (blue, column 2), and LIF-DT neuron spike trains (magenta, column 3). As expected, errors are lower for the reconstructions using the optimal coder. However, fidelity of coding is only one issue. The optimal coder reconstruction also preserves amplitude and timing features of the stimulus unlike the LIF reconstructions which suffer amplitude and phase (time-delay) distortions, and temporal distortions such as lengthening (smearing) of estimated stimulus duration. The quality of reconstructions from the LIF-DT neuron are in-between those of the optimal coder and the LIF reconstructions, with noticeable distortion at higher signal amplitudes and phase distortion at the lowest signal amplitudes. The LIF-DT neuron preserves spike-timing information much like the optimal coder (see Figure 2). These results suggest that information in spike-timing is necessary to recover stimulus features without distortion and without appreciable time-delay. This has consequences for signal detection and signal parameter estimation, and is taken up in detail in the Discussion.

Figure 7 illustrates the effect of reconstruction when using sub-optimal time-constants, for the stimulus at −10 dBV. Reconstructions with three filter time-constants are depicted (A–C), for optimal coder (column 1) and LIF neuron (column 2). Figure 7A uses a small time-constant (11 ms) that is optimal for the LIF neuron (Figure 5A) but not the coder, Figure 7B uses a larger time-constant (22 ms) that is optimal for the optimal coder (Figure 5A) but not the LIF neuron, and Figure 7C uses an arbitrary but large time-constant (45 ms) that is sub-optimal for both coder and LIF neurons. There is appreciable distortion in the reconstructions from the LIF (column 2) even with the best time-constant (Figure 7A2), in particular, the onset and offset times of the reconstruction lag the stimulus. The coder also introduces distortion at large time-constants (Figure 7C1) but with less deleterious effect on the onset time and amplitude. At the lowest time-constant, the coder (Figure 7A1) resembles the smoothed PSTH (see Figure 3B2) but with noticeable ramping of the reconstruction at the stimulus onset (the peak of the reconstruction leads the stimulus). It is likely that spike timing in the optimal coder (which captures timing features of real neurons) leads to robust reconstructions when confronted with a broad range of post-synaptic membrane time-constants. This may not be true for LIF or renewal-type spike trains where reconstructions can suffer distortions when the post-synaptic membrane has a sub-optimal time constant.

Figures 5A, 6, 7, show that the optimal coder formulation does provide high fidelity reconstructions. However, is it possible to generate reconstructions that have much higher fidelity than the optimal coder? The optimal coder proposed here uses a simple first-order low-pass filter, and other filter structures can possibly generate better reconstructions. To demonstrate this we used a non-causal Wiener filter (a minimum mean-squared estimator or MMSE), similar to the estimator proposed by Gabbiani (1996) and Gabbiani and Koch (1996). The experimentally obtained P-type spike-train was used as the input to the Wiener filter, with the stimulus as the desired output. The reconstruction errors were much smaller (waveforms are not shown but are similar to the optimal coder reconstructions shown in Figures 6A1,B1,C1). They are (with stimulus intensity in parenthesis): −13.8 dBV (0 dBV), −14.4 dBV (−10 dBV), and −16.2 dBV (−20 dBV). The corresponding numbers for the optimal coder reconstructions reproduced from above are −10 dBV, −12.7 dBV, and −13.9 dBV, respectively. Thus, the Wiener filter provides about 2-4 dBV improved fidelity. The improved performance with the Wiener filter is due to the large number of degrees-of-freedom (the filter-length, 9217 samples, or 533 ms) allowing it to be tuned optimally. Further, some improvement is also obtained by using a non-causal filter. The Wiener-filter approach can only be used for reconstruction of a signal in a given time window when both signal and spike-times in that window are known. By design, the reconstruction error is minimized over the entire window. For this reason it is often referred to as “reverse estimation.” A change in the signal or the spike-times would result in a different set of filter coefficients. This is not biophysically realizable. On the other hand, the first-order optimum decoding filter is causal with only two degrees-of-freedom (*A* and τ). The filter parameters can be fixed in advance and are not signal-dependent. They are fixed by the energy constraint and hence can be considered intrinsic biophysical parameters. As discussed later, and supported by other research, several biophysical mechanisms can support the optimum decoder.

Changing the average spike-rate (i.e., the energy-constraint) profoundly influences reconstruction error. Intuitively we expect that reconstruction error will reduce with increasing spike rate (for a fixed reconstruction filter time-constant) due to improved signal representation. This is true, and is depicted in Figure 5B as reconstruction error (ordinate) vs. baseline spike rate (abscissa) at stimulus intensity of −10 dBV. It should be noted that the depicted lines are simulations. For a given baseline spike rate, the optimal coder parameters were determined from Equation (6). Then the optimal encoder was stimulated as in Figure 2A at −10 dBV. The procedure was repeated to cover the range of baseline spike rates shown in Figure 5B. The singleton data points are based on experimental data with the errors as reported in Figure 5A. The P-type spike rate is shown as the filled black circle, the matched optimal coder and LIF neuron spike rates are also shown (filled circles) with some discrepancy from the experimental rate (± 15 spikes/s) due to the iterative nature of parameter estimation. As expected, the coding error monotonically decreases with spike rate for the optimal coder and is smaller than the error for the LIF and LIF-DT neurons at any spike-rate. In particular, a reconstruction of the input from the P-type experimental data (black circle) results in an error that lies between the error from the LIF-DT neuron matched to the spike-rate of P-type data (magenta circle) and the optimum coder (red circle). All three reconstructions are within 3 dBV, and significantly lower than the matched LIF neuron (blue circle). The coding-error vs. spike-rate curve shown in Figure 5B represents the energy-fidelity trade-off. It is outside the scope of this work to specify how exactly the operating point of the neuron is selected along this curve. It is presumably regulated by long-term influences from higher levels, or it may be determined by immediate functional requirements. Figure 5B is analogous to the rate distortion function (coding error as a function of bit-rate) used in information theory to study source encoders (Cover and Thomas, 2006). Just as reconstruction error can be minimized by transmitting digital signals at high bit-rates so also are reconstructions improved with a higher spike-rate. The energy-fidelity curve has received insufficient attention in the neuroscience literature but is likely to be a critical parameter when exploring energy-efficient codes.

### Coding in cortical pyramidal neurons of the rat

#### Encoding and spike-timing

Figure 8A shows *in vitro* data from a cortical pyramidal neuron (see Materials and Methods) in response to injected current (frozen noise, top black trace; raster plot of 13 trials), the optimal coder response (red spikes), and matched LIF neuron (blue spikes). Total duration is 21.5 s. Note that we filtered the stimulus (low-pass, with a time-constant of 20.3 ms). Parameters are reported in Materials and Methods. Experimental spike data from Trial 1 (red arrow) was used to tune the optimal coder and LIF neuron with a matched spike rate (energy constraint). Insets B and C, arbitrarily selected (duration 1.5 and 1 s, respectively), are expanded and shown in Figures 8B,C. Broadly, the optimal coder predicts spike-timing with good accuracy, with mean Γ = 0.38 ± 0.02 compared to the 13 experimental trials. Predictability was good for those spikes where inter-trial timing is reliable, but there is greater ambiguity when spike-timing is less reliable (either missing spikes or shifts in the timing, see Figures 8B,C). The LIF spike train exhibited poorer coincidence with Γ = 0.12± 0.02, although it appears to match the cortical and coder spike trains (Figure 8A, blue spikes). However, when seen on an expanded scale (e.g., as in Figures 8B,C), it can be seen that there is poor coincidence reflected in the smaller value of Γ. The LIF-DT neuron also shows comparable performance with the optimal coder, with Γ = 0.38± 0.04. The coincidence for the optimal coder was significantly higher than the LIF model but not the LIF-DT model (Mann–Whitney *U*-test, *p* < 0.01).

#### Decoding and reconstruction error

Figure 9 shows the reconstruction error as a function of reconstruction filter time-constant for optimal coder (red), LIF neuron (blue), LIF-DT neuron (magenta) with fixed spike rate (9.74 spikes/s). This figure and explanations are the same as Figure 5A. Decoders for reconstructing the LIF and LIF-DT neuron are hypothetical post-synaptic decoders because they do not have a built-in decoder. The optimum values of time-constant are 224 ms (optimum coder), 150 ms (LIF neuron), and 187 ms (LIF-DT neuron). The best filter for the experimental cortical spike train is also shown (155 ms, filled black circle). In contrast to the P-type afferent, the reconstruction errors from optimal coder, LIF, and LIF-DT neurons are similar and differ by about 1 dBV. The optimal coder had the lowest reconstruction error (−5.9 dBV), although the LIF-DT (−5.7 dBV) and experimental (−5.3 dBV) reconstructions are similar. This is despite the better spike time-prediction with the optimal coder than with the LIF neuron. The error for optimal decoding is also higher (compare with reconstruction error from Figure 5A). The higher error may be due to the lower average spike rates for cortical cells (9.74 spikes/s).

Optimum reconstructions using the best filter time-constants from Figure 9 are shown in Figure 10. The stimulus trace (black) is overlaid with the reconstructions of the stimulus (optimal coder, red; LIF neuron, blue; LIF-DT neuron, magenta). Figure 10A covers the entire stimulus duration (reconstruction errors are specified above the trace), and Figure 10B shows an expanded view of inset B. Also included in inset B is the cortical spike train (bottom). In Figure 10B, it can be seen that the LIF neuron demonstrates considerable lag when reconstructing sharp transients (e.g., arrows a and b) whereas the optimum coder reconstruction matches the onset transients with almost no lag (Figure 10B, top trace). At first glance this is somewhat puzzling given that the time constant for the LIF neuron (150 ms) is smaller than the time-constant of the decoder built into the optimal coder (224 ms). As a result, the decoder for the LIF spike train should react with smaller time delay than the optimum decoder. However, the decay rate of the reconstruction filter impulse response *h*(*t*) (which is a single time-constant) should not be confused with the complex signal-dependent decay seen between spikes in the optimum decoder. This is made clear by Equations (13) and (14) and Figure 1C. The LIF-DT neuron also fires spikes when the input stimulus is small. These errors lead to higher error encoding. For the optimum decoder, the decay between spikes is signal level-dependent, with a faster decay when the signal amplitude is high. Thus sharply rising transients are captured more quickly by the optimum decoder than by an LIF neuron. The quick response to a rapid attack is also seen in P-type units (Figures 6, 7). Thus, when used for coding error, the signal-dependent threshold *r*(*t*) has the advantage that it can follow temporal changes in the input signal more quickly and with greater fidelity. This is a direct consequence of the optimal coding mechanism, and the energy-fidelity trade-off.

## Discussion

The motivation for this work rests on the following assumptions: (1) A neuron is subject to an energy constraint in the form of a limit on the average spike rate. (2) A neuron must transmit information with the highest possible fidelity for a given average spike rate. (3) The encoding neuron has some idea about the process of decoding. The first two assumptions are not new. Energy-efficient coding is a topic of wide interest (see for e.g., Laughlin, 2001; Niven and Laughlin, 2008; for information theory approaches see Levy and Baxter, 1996; Berger and Levy, 2010) as is coding fidelity, which has been examined from information theoretic and statistical signal processing perspectives (for e.g., see Eggermont et al., 1983; de Ruyter van Steveninck and Bialek, 1988; Gabbiani, 1996). However, little is known about the influence of the energy-fidelity trade-off on the spike-generation process.

### The energy-constrained optimal neural coder

The coding mechanism proposed here addresses the energy-fidelity tradeoff by assuming that a neuron simultaneously monitors the quality of its signal encoding while satisfying an energy criterion. This leads to a firing rule that optimally times spikes to meet the energy-fidelity trade-off. We choose the energy constraint to be the fixed long-term average spike-rate. This is a reasonable choice because spike generation and transmission consume the most significant part of a neuron's energy budget (see Niven and Laughlin, 2008), accounting for 20–50% of the energy consumed by the brain (Laughlin, 2001; Sengupta et al., 2010). It is very likely that the energy constraint in a neuron is imposed in many ways, depending on the species, the type of neuron, etc. [see Sengupta et al. (2010) for energy efficiency of an action potential, and Sengupta et al. (2013) for balancing excitation and inhibition]. However, the biochemical and biophysical processes that constrain energy are not taken into account here because we consider spike rate as the dominant constraint, and simply estimate the rate from the experimental spike data. This is a major simplification and does not necessarily capture all aspects of an energy constraint. Future work should incorporate other constraints, such as those identified by Sengupta et al. (2010, 2013).

The second component of the proposed neural coder is the mechanism for determining coding fidelity (i.e., decoding). The most common motif in neural processing is the pre- and post-synaptic pairing of neurons where the post-synaptic element filters the pre-synaptic spike train and recovers (decodes) the original encoded signal. Thus, the simplest decoder is a post-synaptic membrane that is a leaky integrator. Is it likely that a neuron has a built-in mechanism that mimics post-synaptic filtering at least in a broad sense, i.e., a low-pass filter? If so, it would provide an organism with selective advantage because sensory neurons could monitor coding fidelity through an internal estimate of the coding error. We hypothesize that such an internal decoder *h*(*t*) is similar to the well-known dynamic threshold, also called a moving or adaptive threshold (Figures 1B,C).

The proposed decoding filter should be interpreted with care. We are not suggesting that every neuron “matches” its decoding filter to that of its post-synaptic neuron. Rather, we suggest that the internal decoding filter [the dynamic threshold *h*(*t*)] is a generic mechanism that converged on “low-pass filter dynamics” in a blind fashion, over evolutionary time-scales. The passive cell membrane (lipid bilayer with passive leakage) is a phylogenetically old structure, and was present in the earliest single-celled eukaryotes before the advent of voltage-gated sodium channels (and action potentials). It has most likely remained unchanged since the earliest organisms, and so it is not unreasonable to suggest that the neural threshold dynamics converged to low-pass dynamics. We do not claim that such type of decoding is true everywhere in the nervous system. However, for sensory processing at least, where temporal features of stimuli need to be extracted, a passive membrane is often sufficient for decoding, and we suggest that this was reasonably approximated by natural selection in the form of the threshold impulse response *h*(*t*). To state it in other words, in the language of evolution, generating the decoded signal *r*(*t*) is a proximate mechanism, whereas converging to low-pass threshold dynamics *h*(*t*) is the ultimate mechanism.

The notion of an externally generated error signal is commonly used in predictive coding models of the auditory system (for example Balaguer-Ballester et al., 2009) and in hierarchical prediction in the visual system (for example Rao and Ballard, 1999; Lee and Mumford, 2003). Predictive coding models differ from the proposed approach in several key ways. The predicted input is generated by higher-level brain structures at the network-level and provides descending information to generate a reference signal against ascending sensory information. The proposed neural encoder, however, is conceptually different and does not rely on a descending reference signal from higher-level brain structures. Rather, we propose that individual neurons are capable of tracking encoding error internally, without direct feedback from upstream neurons. A significant advantage of internal decoding is the lack of delay in tracking the coding error because error-tracking can be implemented at the biophysical rather than the network level. The rapid dynamics allow for quick adjustments in spike-timing in response to time-varying stimuli. There is however, no reason to exclude the possibility that predictive signals generated from postsynaptic or higher-level neurons can shape the encoder/decoder response. In fact, it is very likely that tuning of the decoder filter (time-constant, gain, etc.) will be subject to more slowly-varying influences from predictive signals across the network. These can shape coding on time-scales much longer than those considered here.

### The threshold as optimal decoder

The term “threshold” conventionally referred to a fixed value of the membrane potential at which a spike is initiated (typically lying in the range −55 mV and −45 mV). However, the increased refractoriness of neurons to sustained stimuli suggested that the neural threshold may not be a constant, but was influenced by the history of spiking activity. A dynamic threshold (sometimes referred to as an adapting or moving threshold) was originally proposed by Buller et al. (1953) and Hagiwara (1954) as a mechanism for generating anti-correlations in the observed sequence of interspike intervals (ISIs) and adaptation in the spike rate. Numerous forms have appeared in the literature over the years. In weakly electric fish, the model has been used to predict ISI correlations using non-resetting (Brandman and Nelson, 2002) and resetting inputs (Chacron et al., 2003). In recent years, dynamic threshold models using resetting (Jolivet et al., 2004; Brette and Gerstner, 2005) and non-resetting inputs (Kobayashi et al., 2009) have been used to predict spike-timing in neurons with considerable accuracy^4^. These are feed-forward applications used solely for spike generation. Several forms of the threshold are not dependent on the stimulus *s*(*t*) (for example Chacron et al., 2003), but the form considered here is stimulus-dependent much like those proposed by Brandman and Nelson (2002) and Kobayashi et al. (2009). More recent studies of coding and spike initiation favor a stimulus-dependent threshold (see Platkiewicz and Brette, 2010; Fontaine et al., 2014). The work reported here shows that there is an intimate connection between the stimulus and the threshold, and that such a dependency is necessary for optimal coding.

We suggest that in the form used here (see Figure 1B) the threshold is ideally suited to function as a decoder. It can track the input signal by reconstructing it from the spike train, and provide an internal error signal. That is, it can keep track of coding fidelity. The coder then maximizes coding fidelity by producing an optimum firing policy [firing threshold, γ (*s*, *t*)] for timing spikes. The firing threshold is most appropriately interpreted as a bound on the permissible coding error (Figures 1B,C). The error signal manifests as the sub-threshold signal (equivalent to the sub-threshold membrane potential in biological neurons) causing the coder to fire when the error reaches the threshold from below (Figure 1C). For small signals where *s*(*t*) ≪ *A*, where *A* is the reconstruction filter gain as in Equation (5), the firing threshold γ (*s*, *t*) is signal-dependent. In the asymptotic case, when *s*(*t*) ≫ *A* the firing threshold is independent of the signal, and is constant with γ = *A*/2. The signal dependency is highly compressive, i.e., the variation in γ (*s*, *t*) over the entire range *s* > 0 is limited to the range 0.21*A* < γ < 0.5*A* (see Figures 1B,C, **12**). In Figure 1A it can be seen that the neuron fires a spike when the reconstruction error *s*(*t*) − *r*(*t*) reaches the firing threshold. Therefore, the coding error is coupled to the spike generator because it determines when the neuron should fire. Thus, the proposed coder is a non-linear feedback system that tracks and regulates the coding-error. This is the most radical departure from traditional feed-forward neural coding schemes (spike-generators).

The decoding filter determines the long-term average spike-rate of the neuron. As such it is the mechanism that imposes an energy-constraint. We show how the decoder parameters are set to achieve a given long-term spike-rate (Optimum Encoder Formulation). Once the decoder parameters are set, the optimization process and the spike-rate constraint impose a bound on the reconstruction error. To put it simply, maintaining a high spike-rate will lead to smaller coding errors and better reconstructions of the input signal. It is likely that the spike-rate constraint is imposed by cellular and network processes and is dependent on the functional role of the neuron in the circuit. Once the spike-rate is fixed, the product *A*τ is known from Equation (6). Given this single degree-of-freedom, exactly how *A* and τ should be uniquely determined may depend on functional requirements. Once these parameters have been determined, the optimum firing threshold uniquely fixes the optimal spike-times. In contrast, the validation of the optimum coder is a “reverse” problem because the energy constraint (long-term spike-rate) must be determined from experimental data, and the single degree-of-freedom given by the product *A*τ must be resolved. To achieve this, the decoder parameters are set so that: (1) the long-term spike-rate matches the spike-rate from the experimental data, and (2) the optimal coder produces spike-times that have the closest match (coincidence) with the experimental spike-times. This tuning is *ad hoc* and carried out for the sole purpose of matching the optimal coder to the experimental spike train. Once the parameters (*A*, τ) are fixed, the firing threshold γ (*s*, *t*) is optimized (see Optimization Procedure for Comparison against Experimental Data).

The dynamic threshold *h*(*t*) has been proposed, in a broad sense, as evolutionary convergence to low-pass (passive membrane) dynamics. It is not a perfect decoder, nor is it exclusive, and it leaves ample room for other processes to come into play. One possibility is that more complex threshold dynamics can be considered, such as second- and higher-order low-pass filters (Kobayashi et al., 2009), a form such as *A* exp (*c*/*t*) (Hagiwara, 1954), or the linearized form of Hagiwara's threshold (Buller et al., 1953; Brandman and Nelson, 2002). These filter structures may be tuned by descending influences to shape stimulus coding, as discussed later. Another possibility is the use of pre-filtering. While the threshold *r*(*t*) proposed here is most likely located in the trigger zone of the neuron, dendritic and somatic processing can tune the time-scales of the inputs to which the neuron is responsive. For example, low-pass filtering in the dendrites or soma can shape the bandwidth. The optimal coder framework proposed here does not take into account such filtering because the coder has no access to any of the upstream operations that shape *s*(*t*). This is particularly relevant when considering models that use some form of leaky-integration with a known filter *G*(ω) (fourier transform of the impulse response *g*(*t*)). In this case, if the delivered stimulus is *X*(ω) then the sub-threshold membrane potential is given by *S*(ω) = *G*(ω)*X*(ω), where *S*(ω) is the fourier transform of *s*(*t*). The coder encodes *s*(*t*) but has no knowledge of the delivered stimulus *x*(*t*). Linear operations such as these will not introduce anharmonic distortions during reconstruction. However, non-linear operations such as resetting the integrated input (as in the various forms of the LIF neurons with dynamic threshold, such as the LIF-DT neuron, e.g., Chacron et al., 2001, 2003; Liu and Wang, 2001; Jolivet et al., 2004; Brette and Gerstner, 2005) or the use of dynamic threshold with non-linear integrate and fire (IF) models such as the exponential IF neuron (e.g., Fontaine et al., 2014, based on the exponential IF model of Fourcaud-Trocmé et al., 2003) will introduce distortions in the reconstructions and can potentially affect coder performance. We have shown that one consequence of resetting in the LIF-DT model is an increase in the reconstruction error (as compared to the optimal encoder, see Figure 5). The effects of such non-linearities on coding fidelity need to be carefully examined, but are not considered further here. The most appropriate combination of pre-filtering operations or choice of decoding filter parameters will most likely depend on the functional role of the neuron in the given circuit. The proposed coder does not rule any of them out. Our goal is to demonstrate that the threshold can track coding-error and provide internal feedback to the spike generator.

In this work we consider a time-varying threshold at an abstract level by lumping all the currents into a simple adaptation model (a low-pass element). This approach leads to an analytically tractable model but does not explicitly model biophysical mechanisms. Biophysically realistic currents can be incorporated into the threshold dynamics. Benda and Herz (2003) incorporate M-current, AHP currents, and slowly recovering Na currents, while Jolivet et al. (2004) consider a full-conductance model that incorporates an adapting potassium conductance. Liu and Wang (2001) and Benda et al. (2010) provide a comparison of models with dynamic (adapting) threshold and biophysically realistic currents such as those considered by Benda and Herz (2003). Chacron et al. (2001, 2003) suggest that the low-pass dynamics built into their dynamic threshold model may be mediated by Kv3.1 channels, but their model did not specifically incorporate Kv3.1 dynamics. In summary, there is a diversity of approaches in the study of dynamic or adapting thresholds, ranging from the abstract to the biophysically realistic. We hope that this work will spur biophysically realistic refinements to the proposed optimal coder.

Non-resetting dynamic threshold models (Brandman and Nelson, 2002; Kobayashi et al., 2009) use *ad hoc* firing-rules, such as *s*(*t*) − *r*(*t*) = 0, rather than the more general firing policy *s*(*t*) − *r*(*t*) = γ = γ (*s*, *t*) which allows γ to be optimized for coding fidelity. Indeed, the motivation for proposing a general firing policy here is to allow the reconstruction error to be minimized in a principled and optimal way. Further, it makes apparent the close connection between the time-varying threshold and reconstruction. Earlier models that used dynamic thresholds were designed to match spike-times or interval statistics and are not optimized for coding fidelity. They are sub-optimal coders. By imposing an energy-constraint and a fidelity criterion, we can derive an optimum firing policy. This policy optimally times spikes and demonstrates that the threshold is an optimal coder. The LIF neuron exhibits much higher errors (Figures 5, 9), as will any feed-forward spike generator that does not regulate error. Models such as LIF-DT perform better than the LIF neuron provided they are tuned to match the spike-rate and spike-times, as done by Kobayashi et al. (2009). However, the lack of a built-in decoder and the hard non-linearity due to the reset of the integrator following a spike will result in sub-optimal reconstruction (Figures 5, 9) and introduce phase distortions (Figure 6).

While the impulse response of the time-varying threshold *h*(*t*) has simple first-order dynamics, the error signal *s*(*t*) − *r*(*t*), Equation (1), demonstrates multi time-scale behavior. The decay rate of the error signal (between ISIs) is governed by the signal amplitude in addition to the time-constant of the threshold *h*(*t*) (Figure 1C). The time-constant of the threshold is set by the energy-constraint (spike rate), and governs mean spike rate. However, the instantaneous firing rate is further regulated by the amplitude of the time-varying input signal [Equations (13) and (14)] allowing for fine control of the spike-timing. A voltage-dependent conductance with variable time-constant can readily serve to encode such error dynamics. The firing threshold γ is dependent on the value of the input signal *s*(*t*) although this dependence is fairly small (see above for more details, and Figures 1B,C, **12**). In most cases the threshold can be approximated by γ = *A*/2. There is evidence suggesting that the firing threshold may be dependent on the signal-level. Recently for example, Fontaine et al. (2014) showed that spike-thresholds can vary with the level of the membrane voltage. While their model assumes fast adaptation (of the order of 1 ms), and is based on Na-channel inactivation, the time-constants exhibited by *h*(*t*) for the data considered here cover much larger timescales, from 10 to 100 ms. These may be mediated by a voltage-dependent conductance, such as the M-current which is known to contribute to increased refractoriness on short to long time-scales (Brown and Adams, 1980).

### Optimum spike-timing and stimulus coding

The predictions made by the optimal coder are supported by data on spike-timing in peripheral and cortical sensory neurons. Broadly, the optimal coder captures spike timing (Figures 2, 3, 8–10). This is not surprising because time-varying threshold models are known to predict spike timing with good accuracy (Kobayashi et al., 2009). The optimal coder, however, provides a principled way to generate spikes and makes predictions. For example, it provides an explanation for the responses at stimulus onset and offset. The coder is sensitive to rapid changes in the signal, and will fire rapidly for large positive gradients to keep the error within limits (onsets in Figures 2, 3, 10B). Likewise, the best firing policy when the signal decays faster than the reconstruction is to not fire at all (Figure 2B, inset d), otherwise a spike will cause the reconstruction error to increase. Thus, the onset and offset responses seen in the P-type afferent data and in other primary and primary-like sensory neurons (for e.g., Kiang et al., 1965) are a natural outcome of optimal coding, and a consequence of error regulation using feedback (Figure 1A).

Reconstructions from the internal decoder accurately track the time-course of the stimulus without amplitude or phase distortions (Figures 6, 10B, coder, and compare with LIF and LIF-DT). In particular, the onset is tracked without delay (Figure 6) over a range of decoder time-constants (Figure 7) found in P-type post-synaptic neurons (Berman and Maler, 1998). Thus, the optimal coding neuron can perform sequential (real-time) signal detection with minimum delay thereby allowing for the rapid detection of sensory signals (Ratnam and Nelson, 2000; Goense and Ratnam, 2003). This is an ethologically important function across taxa.

The proposed encoder is based on simple assumptions and it is deterministic, but the prediction of spike-times is good even when the spike rate is low, as with the cortical neuron (Figures 8, 10). In particular, input fluctuations between spikes (e.g., Figures 8B,C, 10B) which are not accounted for in the coder do not seriously degrade the predicted spike times. This is presumably due to the energy constraint which implicitly fixes the temporal and amplitude scales to which the decoder is responsive. For example, spikes are almost always preceded by rapid attacks with large amplitudes (see earlier), while small amplitude fluctuations appear to be damped (Figures 8B,C, 10B). This suggests that the time-varying threshold may be matched to the statistical properties of the input (via the error signal, Figure 1C), particularly to the time-scales of behavioral relevance, with the neuron tuning its energy-fidelity trade-off to best suit its function. Recently, Fontaine et al. (2014) showed that a fast threshold adaptation mechanism allows the neuron to respond to spikes arriving on millisecond time-scales but filters out slowly varying voltages that are not relevant. An alternative approach to the one proposed here, based on Bayesian inference, also generates a firing rule where the neuron responds maximally to the arrival of new information, e.g., fluctuations in the input (Deneve, 2008). These and other studies on spike thresholds suggest that the time-scale of relevance can shape the time course of threshold dynamics, so that salient information is transmitted in the bandwidth of interest. Interestingly, the time-scale for information processing as proposed here and in other studies, can be shaped by long-term influences (incorporating prior information, learning, etc.) to alter the desired bandwidth as needed. In doing so, the energy-fidelity tradeoff (Figure 5B) provides a locus of operating points along which the neuron remains energy-efficient without compromising coding quality.

## Conclusions

The optimal coding scheme proposed here is a hypothesis. If validated, it would demonstrate that neural coding is not simply a feed-forward process, but involves error regulation that is tightly coupled to an energy constraint. Spikes, which are a precious commodity, would be judiciously output so as to maximize coding fidelity. This would suggest that a single neuron is a far more reliable coder than has been assumed. We provide preliminary evidence to show that a deterministic firing rule leads to accurate predictions of spike times and the various features of the PSTH. This lends support to the hypothesis, but more work is required to verify and consolidate these ideas, particularly to establish biophysical mechanisms.

A time-varying threshold has been inferred but has never been directly measured (e.g., most recently, Fontaine et al., 2014). The proposed neural coder can be validated only if the threshold can be measured and if its mechanisms can be determined. Biophysical mechanisms that can estimate the error *s*(*t*) − *r*(*t*) (Figure 1C) should demonstrate two key features: (1) they should be closely coupled to metabolic processes so that firing rate can be brought under metabolic control, and (2) they must be present in the trigger zone so that they can regulate the firing threshold. There are candidate mechanisms that satisfy these criteria. For instance, the KCNQ/Kv7 (M-current) family of channels (Brown and Adams, 1980) may be likely candidates: (1) They are a regulator of neuronal excitability, and are coupled to metabolic processes via the membrane phospholipid PI(4,5)P_2_ (Delmas and Brown, 2005). (2) They are present in the axonal initial segment where spike initiation takes place (Pan et al., 2006). (3) They have a variable time-constant (approximately 10–100 ms) making it ideal for adjusting ISI timing in response to the error signal, are open at supra-threshold values of the membrane potential, and do not inactivate. These factors make them suitable initial candidates for further investigation. Other types of currents may also be involved at the trigger zone and can govern threshold adaptation over millisecond time-scales to much longer time-scales (across several ISIs). These will further influence the time-scales of coding.

## Mathematics and equations

Consider a reconstruction filter that resembles the post-synaptic membrane of a receiver neuron. The simplest form is a *RC* element (first-order low-pass filter) with impulse response given by

(12) h(t)=Aexp(−αt), t>0.

We assume that this filter is internal to the optimal coding neuron (Figure 1A). The energy-constraint (spike rate) is determined from the amplitude *A* and time-constant τ = α^−1^. Let the coder output a spike at *t* = 0 then the decoded signal is of the form (Figure 1B)

(13) r(t)=C(s)exp(−αt), t>0,

where *C*(*s*) is a function depending on the input signal *s*(*t*). Spikes are fired according to Equation (1) when *s*(*t*) − *r*(*t*) = γ.

**Figure 11:**
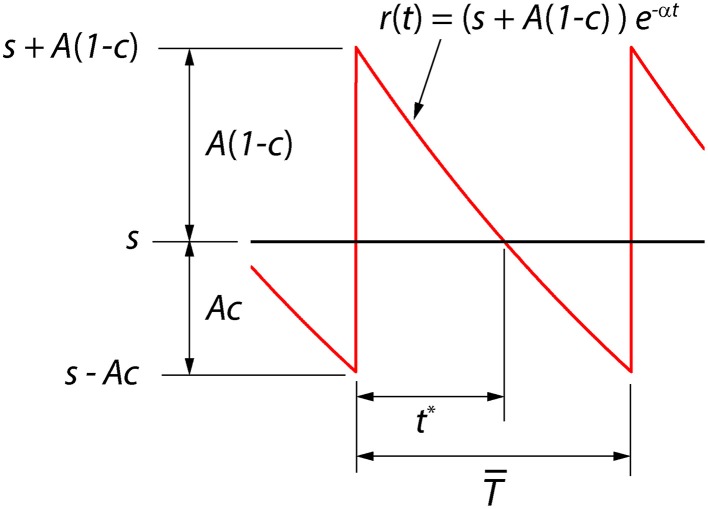
**Geometrical interpretation of the optimization procedure that will perform a minimum-error reconstruction** ***r*****(*****t*****) of a signal** ***s*****(*****t*****) given a constraint on the mean firing rate (*****T*****^−1^)**. The signal *s* (black line) is assumed to be constant within the time-scale of interest (between adjacent spikes). The reconstruction *r*(*t*) (red) is a single-pole low-pass filter with parameters *A* and α. It suffers a discrete jump of magnitude *A* whenever the encoder emits a spike. The decoder then relaxes until it reaches a threshold of *s* − *Ac* whereupon the encoder emits a spike. For fixed *s*, *A*, *c*, and α, the interspike interval is *T* (see text). The parameters *A* and α are known, and determined from a constraint on *T*, and *s* is the instantaneous level of the signal and is a free variable. Only *c* is to be determined so that the error between *r* and *y* is minimized. Here we optimize the value of *c* so as to minimize the mean-squared value of the reconstruction error *s*(*t*) − *r*(*t*). Note that the reconstruction *r*(*t*) is signal level-dependent and carries with it a history of prior spiking activity. Thus, the threshold value of the reconstruction error (Ac) will in general be signal level-dependent. See text for details on the optimization procedure.

Critically, we assume that *s*(*t*) changes much more slowly than *r*(*t*) and therefore remains approximately constant with value *s* between spikes (Figure 11). This simplifying assumption is valid at high spike-rates, when the time between spikes is small. As the inter-spike interval shrinks, the signal can be approximated with decreasing error by a piece-wise constant approximation. This error is bounded by Taylor's Theorem, and the approximation error approaches zero as the spike-rate approaches infinity. Using this simplifying assumption, it is possible to derive an expression for γ which is valid in the limit of high spike-firing rates.

Now, we assume the form

(14) C(s)=s+A(1−c),

where 0 ≤ *c* ≤ 1 is unknown and yet to be determined, and is perhaps dependent on some or all of *s*(*t*), *A*, α, and *y*. The parameter *c* positions the relaxing exponential with respect to the signal *s* (Figures 1B, 11), and the goal is to ask what value of *c* will produce the smallest mean-squared reconstruction error *s*(*t*) − *r*(*t*).

We assume that *s* > *Ac*; otherwise the encoder will never fire. At time *t* = 0^−^, let *r*(0^−^) = *s* − *Ac*. This is the threshold for firing a spike. Note that the threshold is reached from above by *r*(*t*), and at all times *r*(*t*) ≥ *s* − *Ac*. The encoder emits a spike at *t* = 0. Instantly, the decoder suffers a finite jump of magnitude *A* so that at *t* = 0, *r*(0) = *A*(1 − *c*) + *s* = *C*(*s*). If the condition *r*(*t*) = *s* − *Ac* is not met, the encoder holds at zero (does nothing). At *t* = *T*, the decoder will relax to the value *r*(*T*) = *s* − *Ac* and the encoder will fire the next spike. The parameter *T* (mean interspike interval) is the energy-constraint because it determines the mean spike output over time. It can be obtained from experimental data (for example, from the mean long-term firing rate). The parameters of the decoder, *A* and α, are determined from *T* (see further below).

The total excursion by *r*(*t*) in time *T* (between spikes) is *A*. Let ε = *s*/*A*, with ε > *c*, we have

(15) $$\bar{T}=-\frac{1}{\alpha }\log_{e}\frac{\varepsilon -c}{1+\varepsilon -c}.$$

In the following treatment we consider only a single time interval between two spikes, separated by time *T*. We will determine the optimum value of *c* that minimizes the mean-squared error between *r*(*t*) and *y*. Let *E* denote the mean-squared reconstruction error in the interval (0, *T*). That is,

(16) $$\begin{array}{l} E=\int_{0}^{\bar{T}}(s(t)-r(t){)}^{2}\;dt\\ \;=A^{2}\int_{0}^{\bar{T}}((1+\varepsilon -c)\exp(-\alpha t)-\varepsilon {)}^{2}\;dt, \end{array}$$

where we have made use of the substitution *r*(*t*) = *A*(1 + ε − *c*) exp (−α*t*). Substituting for *T* from Equation (15) and carrying out the integration we obtain

(17) $$E={\frac{A}{2\alpha }}^{2}\left\{1-2\varepsilon -2c-2\varepsilon^{2}\log_{e}\frac{\varepsilon -c}{1+\varepsilon -c}\right\}.$$

All terms on the right side except for *c* are fixed. Taking the derivative of *E* with respect to *c* we obtain

(18) $$\frac{dE}{dc}={\frac{A}{\alpha }}^{2}\left\{\frac{\varepsilon^{2}}{\varepsilon -c}-\frac{\varepsilon^{2}}{1+\varepsilon -c}-1\right\}.$$

Setting *dE*/*dc* = 0, and noting that *A* > 0, α > 0, and ε > *c*, we obtain a quadratic equation for *c*

(19) $$f(c)=c^{2}-(1+2\varepsilon )c+\varepsilon =0.$$

Due to the constraint 0 ≤ *c* ≤ 1 only one root is a valid solution, and this is

(20) $$c=\frac{(1+2\varepsilon )-\sqrt{1+4\varepsilon^{2}}}{2}.$$

The second derivative *dE*^2^/*dc*^2^ is

(21) $$\frac{d^{2}E}{dc^{2}}=\frac{A^{2}\varepsilon^{2}}{\alpha }\left\{\frac{1}{{\left(\varepsilon -c\right)}^{2}}-\frac{1}{{\left(1+\varepsilon -c\right)}^{2}}\right\}.$$

At the extreme value given by Equation (20), *d*^2^ *E*/*dc*^2^ = 1 + 4 ε^2^ > 0. Hence Equation (20) minimizes *E*.

Thus, given that the neuron fired a spike at *t* = 0, and the signal varies more slowly than *h*(*t*), the time to the next spike will be given by the firing rule

(22) $$s-r(t)=Ac=A\left\{\frac{(1+2\varepsilon )-\sqrt{1+4\varepsilon^{2}}}{2}\right\}.$$

More generally, we can define a continuous threshold function

(23) γ=f(s,t)=Ac,

where *c* is the instantaneous signal- and time-dependent function given by Equation (20). This leads to the firing rule given in Equation (22).

**Figure 12:**
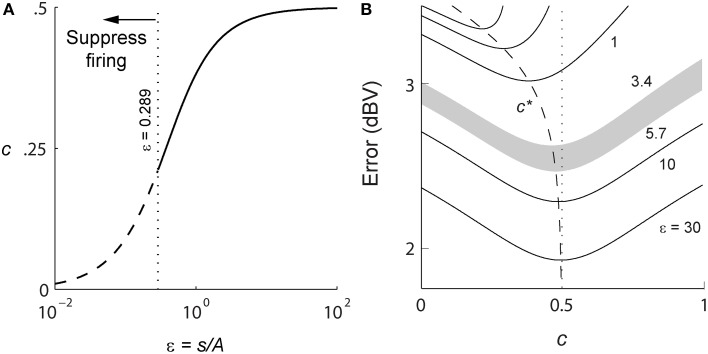
**Optimal coder threshold parameter and reconstruction error. (A)** Threshold parameter *c* (ordinate) as a function of normalized amplitude ε = *s*/*A* (abscissa) showing the wide range of ε over which the asymptotic value of the threshold (c = 0.5) holds. As ε approaches 1, *c* decreases rapidly. When the signal is small, such that ε <1/$\sqrt{12}$ = 0.289 (*c* ≤ 0.21), the optimum policy is to suppress firing because the reconstruction error upon firing a spike will be larger than the error from not spiking. **(B)** Reconstruction error (ordinate, in dBV) as a function of threshold parameter *c* (abscissa) parameterized by the amplitude ratio ε. The locus of optimum values c^*^ for which error is a minimum is shown by dashed line. The large signal asymptote (c = 0.5) is shown as dotted line. The shaded region indicates the values of ε between the 10th and 90th percentile for the noise stimulus shown in Figures 8, 10. In this range the optimum c^*^ lies between 0.464 ≤ *c* ≤ 0.478.

We make the following observations:

1. The optimum value of *c* that minimizes the reconstruction error will depend only on ε and no other parameter.
2. The function *f*(*c*) is a strictly monotone decreasing function in [0, 1]. Further, at *c* = 0, *f* = 1, and at *c* = 1, *f* = −1. This implies that *f* has a real root in the interval [0, 1], and this is given by the solution Equation (20). Further, for large ε, *f* is approximately a straight line with slope of −2. Using this asymptotic approximation we have $\underset{\varepsilon \to \infty }{\lim}c=1/2$ (Figure 12A).
3. When *A* is comparable to *s*, that is when ε ≈ 1 the optimum value of *c* deviates considerably from 1/2. For example, when ε = 1, *c* = (3−$\sqrt{5}$)/2 = 0.382 (Figure 12A).
4. Figure 12B depicts the error function *E* (in dBV) as a function of *c* which is parameterized by ε. The locus of points joining the minima of the curves (optimum *c*) is shown (dashed line) with the asymptote (dotted line). Deviations of the optimum value of *c* are noticeable at values of ε that are smaller than about 10. The shaded area between the curves 3.4 ≤ ε ≤ 5.7 denotes the range of ε values used to stimulate the cortical pyramidal neuron depicted in Figures 8, 10.

Figure 1B depicts the signal *s*(*t*) and the reconstruction *r*(*t*) along with the spike times. The threshold is depicted as a function γ = γ(*s*, *t*) that forms the lower envelope of *r*(*t*). The encoder fires when *r*(*t*) hits this envelope. This is a form of a non-resetting adapting threshold (Brandman and Nelson, 2002; Kobayashi et al., 2009). Figure 1C depicts the reconstruction error *s*(*t*) − *r*(*t*) as a function of time. Viewed in this way, the threshold is shifted so that it has a baseline given by −γ = −*Ac*, where *c* is also a function of signal level *s*, as specified by Equation (20). In contrast with the representation in Figure 1B, the firing threshold is here viewed as a bound on the error *s*(*t*) − *r*(*t*). We conjecture that it is this error signal that has a biophysical analog in real neurons, and which drives the spike generator (encoder) (see also Figure 1A).

If the signal is very small, almost approaching zero (ε → 0) then *c* → 0, and the coder could put out an occasional spike over long durations. However, this may not be desirable because the best reconstruction of a vanishingly small signal may be to not fire a spike at all, and to let the decoder decay freely down to 0. Thus there is an upper-bound on ε beyond which the optimal policy is to not spike. This bound is determined by comparing the reconstruction error if a spike is fired vs. the reconstruction error assuming no spike is fired.

Let the reconstruction error *E* following a spike be specified as before by Equation (16). We define *E*_0_ as the error in reconstruction if no spike was output at *t* = 0, with the decoder going into a free decay. *E*_0_ is given by

(24) $$E_{0}=\int_{0}^{\bar{T}}(s(t)-s(0)e^{\left(-t/\tau \right)}{)}^{2}\;dt,$$

where *r*(*t*) is as given by Equations (13) and (14). We are interested in determining the value of ε where *E* = *E*_0_. This is a cumbersome problem but a good approximation is as follows. We note that *r*(*t* = *T*) = *s* − *Ac* < *s*, and if no spike is fired then *r*(*t*) will further decay and become smaller. Further, *s*(*t*) by assumption is a small signal (ε is small). Thus we set *r*(*t*) = 0 and this simplifies the calculations. This yields

(25) $$E_{0}=\int_{0}^{\bar{T}}s^{2}(t)\;dt.$$

Given that *s*(*t*) = *s*, we obtain from Equations (15) and (25)

(26) $$E_{0}=s^{2}\bar{T}=-\frac{s^{2}}{\alpha }\log_{e}\frac{\varepsilon -c}{1+\varepsilon -c}.$$

From Equations (17) and (26) this yields

(27) $$\frac{1}{2\alpha \varepsilon^{2}}(1-2c-2\varepsilon )=0.$$

Substituting for *c* from Equation (20) we obtain

(28) $$\sqrt{1+4\varepsilon^{2}}-4\varepsilon =0,$$

which simplifies to

(29) $$\varepsilon =\frac{1}{\sqrt{12}}=0.289.$$

For values of ε smaller than Equation (29) the neuron should not fire a spike. From Equation (20) this limit corresponds to *c* = 0.21. So the optimal coder fires a spike only in the region

(30) ε>0.289 or equivalently, 0.21<c<0.5,

and suppresses firing otherwise. Figure 12A shows the functional relationship between *c* and ε. The function is highly compressive (note the log scale of the abscissa). As ε → ∞ we have the asymptotic result *c* → 0.5. This is a useful approximation over a fairly wide range of ε approaching 1, and suggests that the error is robust to changes in *c*. It is a particularly good approximation for the P-type neurons in electric fish that are reported here. They have a high baseline rate of firing. Figure 12A also shows the region along the ε-axis where subthreshold signals will not elicit a spike. The bound provided by Equation (29) is an approximation, and the real bound is likely to be somewhat higher.

We have illustrated the optimization problem and the determination of the optimum firing rule using a simple reconstruction filter with a slowly-varying signal that is constant within an interspike interval. This is admittedly a simplification. For arbitrary signals, with more general forms of the filter, the optimization problem specified by Equation (2) is in general difficult and may not lead to closed-form solutions such as Equation (22). Further work is needed to determine the influence of various forms of the low-pass filter and signal bandwidth on the shift in optimum spike-timing.

### Conflict of interest statement

The authors declare that the research was conducted in the absence of any commercial or financial relationships that could be construed as a potential conflict of interest.

## Footnotes

^1^The description of a membrane element as a passive *RC* element has a long history going back at least to Lapicque (1907). It was elaborated on experimentally and theoretically by a number of investigators in the first half of the twentieth century, including Kenneth Cole, John Eccles, Alan Hodgkin, Bernard Katz, Wilfrid Rall, Nicolas Rashevsky, among others. In its most general form it is a partial differential equation.

^2^www.incf.org/community/competitions/archive/spike-time-prediction/2009/challenge-a

^3^http://www.incf.org/community/competitions/archive/spike-time-prediction/2009

^4^The multi-timescale adaptive threshold (MAT) model (Kobayashi et al., 2009) made the best spike-time predictions in the International Neuroinformatics Coordinating Facility (INCF) 2009 Spike Time Prediction Challenge. See also Gerstner and Naud (2009) for more information.
